# Distinct Patterns of Desynchronized Limb Regression in Malagasy Scincine Lizards (Squamata, Scincidae)

**DOI:** 10.1371/journal.pone.0126074

**Published:** 2015-06-04

**Authors:** Aurélien Miralles, Christy A. Hipsley, Jesse Erens, Marcelo Gehara, Andolalao Rakotoarison, Frank Glaw, Johannes Müller, Miguel Vences

**Affiliations:** 1 Centre d’Ecologie Fonctionnelle et Evolutive, Centre National de la Recherche Scientifique, Montpellier, France; 2 Division of Evolutionary Biology, Zoological Institute, Technical University of Braunschweig, Braunschweig, Germany; 3 Museum für Naturkunde, Leibniz-Institut für Evolutions und Biodiversitätsforschung, Berlin, Germany; 4 University of Melbourne, School of BioSciences, Melbourne, Victoria, Australia; 5 Biosystematics Group, Wageningen University, Droevendaalsesteeg, Wageningen, The Netherlands; 6 Centro de Biociencias, Universidade Federal do Rio Grande do Norte, Natal, RN, Brazil; 7 Département de Biologie Animale, Université d’Antananarivo, Antananarivo, Madagascar; 8 Zoologische Staatssammlung München, München, Germany; National & Kapodistrian University of Athens, Faculty of Biology, GREECE

## Abstract

Scincine lizards in Madagascar form an endemic clade of about 60 species exhibiting a variety of ecomorphological adaptations. Several subclades have adapted to burrowing and convergently regressed their limbs and eyes, resulting in a variety of partial and completely limbless morphologies among extant taxa. However, patterns of limb regression in these taxa have not been studied in detail. Here we fill this gap in knowledge by providing a phylogenetic analysis of DNA sequences of three mitochondrial and four nuclear gene fragments in an extended sampling of Malagasy skinks, and microtomographic analyses of osteology of various burrowing taxa adapted to sand substrate. Based on our data we propose to (i) consider *Sirenoscincus* Sakata & Hikida, 2003, as junior synonym of *Voeltzkowia* Boettger, 1893; (ii) resurrect the genus name *Grandidierina* Mocquard, 1894, for four species previously included in *Voeltzkowia*; and (iii) consider *Androngo* Brygoo, 1982, as junior synonym of *Pygomeles* Grandidier, 1867. By supporting the clade consisting of the limbless *Voeltzkowia mira* and the forelimb-only taxa *V*. *mobydick *and *V*. *yamagishii*, our data indicate that full regression of limbs and eyes occurred in parallel twice in the genus *Voeltzkowia* (as hitherto defined) that we consider as a sand-swimming ecomorph: in the *Voeltzkowia* clade sensu stricto the regression first affected the hindlimbs and subsequently the forelimbs, whereas the *Grandidierina* clade first regressed the forelimbs and subsequently the hindlimbs following the pattern prevalent in squamates. Timetree reconstructions for the Malagasy Scincidae contain a substantial amount of uncertainty due to the absence of suitable primary fossil calibrations. However, our preliminary reconstructions suggest rapid limb regression in Malagasy scincids with an estimated maximal duration of 6 MYr for a complete regression in *Paracontias*, and 4 and 8 MYr respectively for complete regression of forelimbs in *Grandidierina* and hindlimbs in *Voeltzkowia*.

## Introduction

Malagasy scincines represent an ecologically and morphologically diverse lizard clade of approximately 60 recognized species. They likely originated from an African ancestor that reached Madagascar via overseas rafting in the Cenozoic, ca. 60–45 mya [1]. These lizards successfully colonized most of the terrestrial ecosystems of the island and their diversification was accompanied by adaptations to a variety of distinct environments. Most remarkable are the various species that adapted to fossorial habits by several important morphological transformations, such as elongation of the body, miniaturization, loss of pigmentation, regression of eyes and/or external ear openings, and regression or loss of limbs (cf. [2–8]).

Transitions from a fully quadrupedal, lizard-like body form to an almost or completely legless, elongate, snakelike body form have repeatedly occurred in several clades of Squamata (e.g., Serpentes, Amphisbaenia, Scincidae, Anguidae, Dibamidae, Pygopodidae, Cordylidae) and in one clade of Lissamphibia (Gymnophiona), many of these taxa being fossorial. These transformations have apparently occurred progressively, involving first a decrease in the length of limb elements and a reduction of the number of digits and/or phalanges, and an increase in relative body length and number of vertebrae [9–16]. Such a repeated and progressive evolutionary transformation predicts the existence of a variety of intermediate forms among extant species.

**Fig 1:**
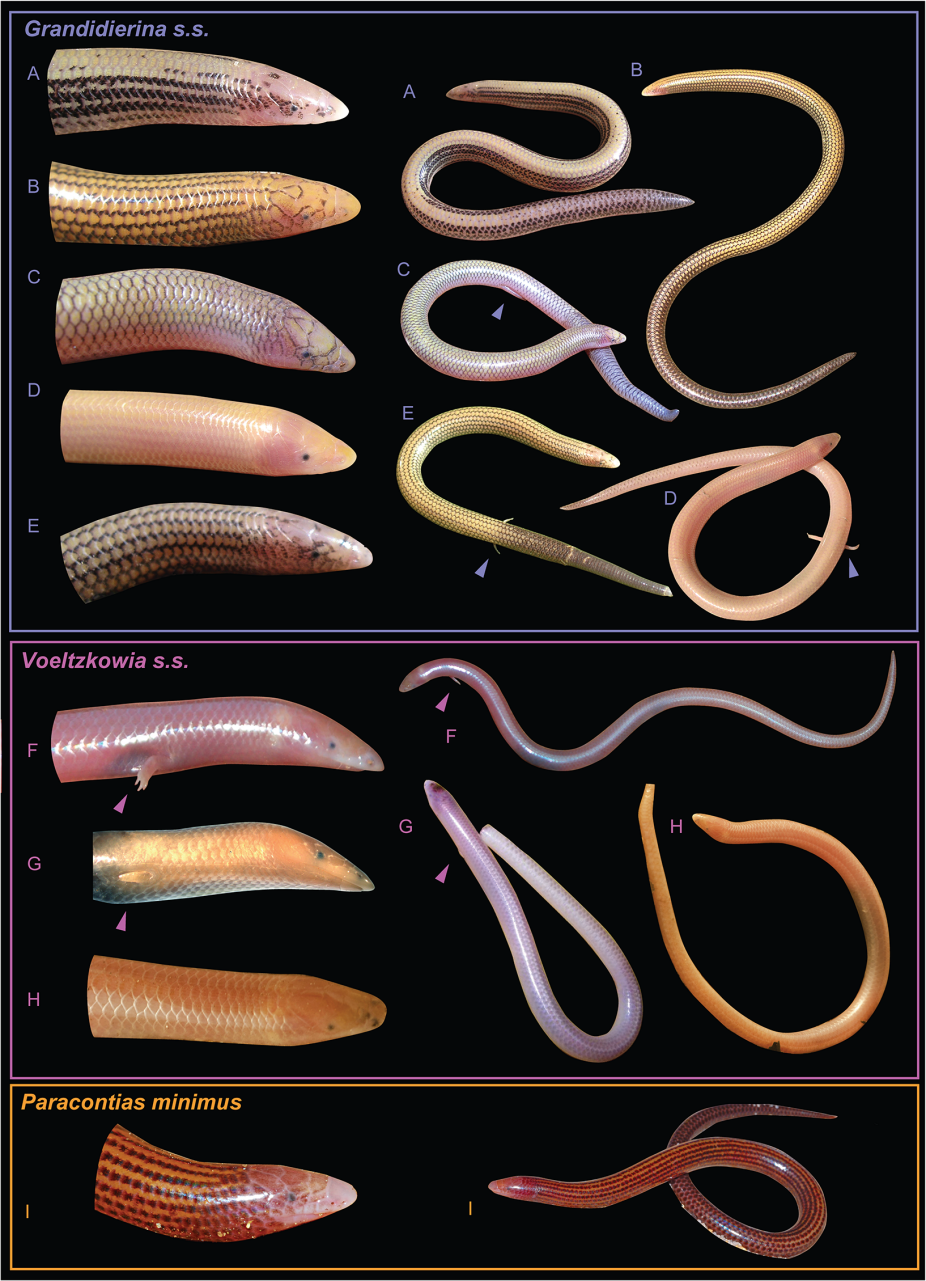
Convergent external morphology of the three clades of Malagasy skinks of the sand-swimming ecomorph. Photographs of (A) Grandidierina rubrocaudata* from Sakabera, (B) *G*. lineata* from Faux Cap, (C) *G*. fierinensis*, dark phenotype from Tombohina, and (D) pale phenotype from Anakao, (E) *G*. petiti* from Sakabera, (F) Voeltzkowia yamagishii** from Ankarafantsika, (G) *V*. mobydick** from the Bongolava plateau, (H) *V*. *mira* from Antsanitia, and (I) *P*. *minimus* from the baie de Sakalava. Arrows indicate limbs. All except G represent living specimens. Photographs from A. Miralles, F. Eckhard, A. Rakotoarison and J. Köhler. * Species included in the genus *Voeltzkowia* until the present study; ** Species included in the genus *Sirenoscincus* until the present study (see taxonomic accounts in Discussion).

Among the 16–20 independent events of complete limb loss recorded in the Scincidae [8], several of the most radical adaptations are found in subterraneous Malagasy taxa [1]. These species, belonging to the genera *Paracontias*, *Sirenoscincus* and *Voeltzkowia*, are small with a midbody diameter less than 5 mm, elongated and slender, almost worm-like, and with complete or partial (anterior and/or posterior pairs) loss of limbs. Despite these similarities, two ecomorphotypes [17], [18] can be distinguished among these small-sized skinks [19], which we here define roughly as sand-swimmers and litter-burrowers: (i) Sand-swimmers (*Paracontias minimus* and species in the genera *Sirenoscincus* and *Voeltzkowia*) are relatively fast moving according to our field observations; they preferentially live in relatively warm, insolated, and dry sandy substrates, such as littoral or riparian dunes, or xeric sandy forests, and are only known from lowland habitats <600 m above sea level. Their skin is partially or even completely unpigmented (light coloration), their eyes are extremely regressed (without recognizable lenses and eyelids) and completely sunken below cephalic scales, and the tip of their snout is conical or spade-shaped (Fig 1). They are legless, or they have either their front or hindlimbs completely regressed. (ii) Litter-burrowers (all *Paracontias* species except *P*. *minimus*) preferentially live in relatively fresh and moist organic substrates, such as leaf litter or under rotten logs in mesic forest from sea level up to over 1000 m asl, although a few species (*P*. *fasika*, *P*. *rothschildi*) also occur in coastal sand. Their skin is usually relatively well pigmented, their eyes are apparently normal or only moderately regressed (always with recognizable lenses and eyelids and never completely sunken under the skin as in the previous group), the tip of their snout is rounded and they are relatively slow-moving and always legless. Additionally, several groups of larger-sized skinks with partial or complete limb-loss exist in Madagascar which might be roughly assigned to these groups but are not in the focus of the present study, i.e., *Pseudoacontias* being litter-burrowers and *Pygomeles* fitting the sand-swimming ecomorph, although with largely normal eyes.

Remarkably, among the Malagasy small-sized sand-swimmers, the limb reduction process appears to be decoupled between fore- and hindlimbs, affecting more rapidly one or the other pair of limbs: (i) The only sand-swimmer with regressed eyes within *Paracontias*, *P*. *minimus*, is completely legless as are its litter-burrower congeners [20]. (ii) The genus *Voeltzkowia* as currently understood shows different morphologies [21], either with two reduced hindlimbs (subgenus *Grandidierina* with two clawed toes in *V*. *fierinensis* or without distinct toes in *V*. *petiti*) or completely legless (subgenus *Voeltzkowia* with *V*. *lineata*, *V*. *mira* and *V*. *rubrocaudata*. (iii) *Sirenoscincus* is again different, having forelimbs only: highly regressed with clawed fingers in *S*. *yamagishii*, or without any distinct fingers in *S*. *modydick* [6], [8].

Molecular phylogenetic studies have demonstrated that regression and loss of limbs convergently occurred numerous times within the Malagasy scincine radiation [1], [22], [23], and at least twice among sand-swimmers (in *Paracontias minimus* and in *Voeltzkowia*), but the relationships of *Sirenoscincus* remain elusive since no molecular data on this genus, nor on the type species of *Voeltzkowia* (*V*. *mira*) have so far become available. Important morphological similarities between both *Sirenoscincus* and *Voeltzkowia* suggest possible close phylogenetic relationships among at least some representatives of the two genera, but due to the mosaic of plesiomorphic and apomorphic traits identified in both taxa in a previous study [8], their relationships remained elusive.

Due to their variation in limb regression patterns, Malagasy skinks of the sand-swimming ecomorph represent a remarkable model to study extreme morpho-anatomical changes modifying the classical tetrapodal vertebrate bodyplan. Here we contribute to such an analysis by providing (i) a multilocus molecular phylogeny of Madagascar's skinks with an expanded sampling focused on species of the sand-swimming ecomorph, and (ii) a comprehensive microtomographic data set with which to examine and compare appendicular skeleton anatomy of these different “blind sand-swimming” taxa. We combine these analyses with (iii) a more detailed population genetic analysis of the polymorphic species *Voeltzkowia fierinensis*. The closer look at this species was motivated by the need of assessing the correct number of species-level units for ancestral character state reconstruction, and by the opportunity to better understand the potential for fast morphological evolution in Malagasy skinks by studying the polymorphic *V*. *fierinensis*.

## Materials and Methods

### Ethics statement

No experiments were conducted using live animals. All field research, collecting of specimens, including in situ euthanasia of specimens were approved by the Madagascan Ministère de l’Environnement, des Eaux et des Forets (Direction des Eaux et Forets, DEF) under the following permits: 156-MEF/SG/DGEF/DADF/SCB dated 12 December2002; 238MINENVEF/SG/DGEF/DPB/SCBLF dated 14 November 2003; 238MINENV.EF/SG/DGEF/DPB/SCBLF/RECH dated 22 December 2004; 272MINENV.EF/SG/DGEF/DPB/SCBLF/RECH dated 8 November 2005; 298MINENV.EF/SG/DGEF/DPB/SCBLF/RECH dated 22 December 2006; 036/08 MEEFT/SG/DGEF/DSAP/SSE dated 30January; 2008;26/09/MEEFT/SG/DGEF/DSAP/SLRSE dated3 February 2009; 48/09/MEEFT/SG/DGEF/DSAP/SSE dated9 March 2009; 188/09/MEEFT/SG/DGEF/DSAP/SSE dated 16 September 2009; 195/09/MEEFT/SG/DGEF/DSAP/SSE dated 28 September 2009; 314/10/MEF/SG/DGF/DCB.SAP/SCB dated 4 November 2010, and 232/12/MEF/SG/DGF/DCB.SAP/SCB dated September 4, 2012. Export of specimens was approved by the DEF under permits: 063C-EA02/MG03, dated 26 February 2003; 094C-EA03/MG04, dated 1 March 2004; 103C-EA03/MG05, dated 15 March 2005; E1400/06, dated 1 June 2006; 055N-EA03/MG10, dated 25 March 2010. Voucher specimens were euthanized using approved methods (e.g. anesthesia with ketamine, followed by ketamine overdose and 95% ethanol fixation).

### Taxonomy

Anticipating our taxonomic conclusions, we herein (except for the Introduction) consistently use the genus names (i) *Grandidierina* for species in south-western Madagascar previously considered as *Voeltzkowia*, (ii) *Voeltzkowia* for three north-western species, including two previously in the genus *Sirenoscincus*, and (iii) *Pygomeles* for the two species previously in this genus plus *P*. *trivittatus* which previously was classified in *Androngo*. Genus abbreviations are *A*. for *Amphiglossus*, *An*. for *Androngo*, *G*. for *Grandidierina*, *M*. for *Madascincus*, *Pa*. for *Paracontias*, *Ps*. for *Pseudoacontias*, *Py*. for *Pygomeles*, *S*. for *Sirenoscincus* and *V*. for *Voeltzkowia*.

### Samples, specimens and morphology

For the molecular analyses, 74 samples of Malagasy skinks were collected across Madagascar between 2001 and 2010. Specimens were sacrificed using ketamine anaesthesia and overdose, a piece of tissue was removed and stored in 95–99% ethanol, and voucher specimens were fixed in 5% formalin or 95% ethanol and stored in 70% ethanol. We examined the morphology of a total of 108 preserved specimens, including most type specimens of the sand-swimming ecomorph, from the Muséum National d’Histoire Naturelle, Paris (MNHN); Museo Regionale di Scienze Naturali, Torino (MRSN); Natural History Museum, London (NHM); Université d'Antananarivo, Département de Biologie Animale (UADBA), and Zoologische Staatssammlung München (ZSM). FGZC, FGMV, MV, and MgF refer to Frank Glaw, Miguel Vences and Madagascar Frontiers field numbers. For a list of all voucher specimens examined, with geographical coordinates of collecting localities see S1 Table. Meristic, mensural and qualitative characters examined here are those routinely used in the taxonomy of Scincidae [24]. See Miralles *et al*. [7], [8], [25], [26] for details on the scale nomenclature used.

### Anatomy

Microtomographic data were generated to assess the appendicular skeleton anatomy for most species of the sand-swimming ecomorph: all four species of *Grandidierina*, two of the three *Voeltzkowia*, and *Paracontias minimus;* and for *Amphiglossus ornaticeps*, a small-sized scincine related to *Grandidierina*, allegedly presenting the plesiomorphic state for Malagasy scincine morphology, with four functional pentadactyl limbs. A single specimen per species was scanned: *G*. *fierinensis* (ZSM 1636/2010), *G*. *lineata* (ZSM 1624/2010), *G*. *petiti* (ZSM 1620/2010), *G*. *rubrocaudata* (ZSM 1632/2010), *V*. *mira* (ZSM 867/0), *V*. *mobydick* (UADBA R70487), *A*. *ornaticeps* (ZSM 1603/2010 = ZCMV 13010) and *P*. *minimus* (ZSM 2253/2007). Scanning took place at the Museum für Naturkunde Berlin using a Phoenix|x-ray nanotom (GE Sensing & Inspection Technologies GmbH, Wunstorf, Germany) with a 180 kV high-power nanofocus tube and a tungsten target. Reconstructions were performed in datos|x-reconstruction software (GE Sensing & Inspection Technologies GmbH phoenix|x-ray) and data were visualized in VGStudio Max 2.0 (Volume Graphics GmbH, Heidelberg, Germany). The upper and lower body of the specimen were scanned separately for 1000 projections each, resulting in a magnification ratio of 5.5x and 6.4x, and a voxel size of 9.2 μm and 7.8 μm, respectively. To visualize skeletal features in three dimensions, such as the pectoral and pelvic girdles, the osteoderms were digitally isolated and rendered transparent. For the remaining elements of regressed limbs we formulated tentative homology hypotheses based on their relative position and size, although embryological evidence have shown that digit identity frame shift may occur as a consequence of digit reduction in skinks [27]. Original pictures published in the present paper have been deposited in Morphobank (available at www.morphobank.org) under the project number 1195 (n = 49 high definition pictures numbered from M332080 to M332129).

### Molecular analyses

#### DNA sequences

We collected DNA sequence data for three fragments of mitochondrial (mtDNA) genes: NADH-dehydrogenase subunit 1 (ND1) with adjacent tRNAs (tRNAMet, tRNAGln and tRNAIle genes), 12SrRNA (12S) and 16S rRNA (16S), and for four protein-coding nuclear genes (nDNA): brain-derived neurotrophic factor (BDNF); recombination activating gene 2 (RAG2); oocyte maturation factor (CMOS) and phosducin (PDC). Standard polymerase chain reactions were performed in a final volume of 12.5 μl containing 0.3 μl each of 10 pmol primer, 0.25 μl of total dNTP 10 mM (Promega), 0.1 μl of 5 U/ml GoTaq, and 2.5 μl of GoTaq Reaction Buffer (Promega). Primers and PCR conditions followed Crottini *et al*. [1]. The successfully amplified products were purified using the ExoSAP-IT purification kit according to the manufacturer’s instruction. Purified PCR templates were sequenced using dye-labeled dideoxy terminator cycle sequencing on an ABI 3130 automated DNA sequencer. The data matrix is 94.3% complete. Sequences were aligned using the ClustalW algorithm and subsequently refined manually in BioEdit 7.0 [28]. We used GBLOCKS [29] with stringent settings (no gaps allowed) to determine and exclude uncertain positions in the alignment. A total of 249 newly determined sequences were deposited in GenBank under accession numbers KM057077 to KM057324 (see S1 Table for a detailed list).

#### Phylogenetic analyses

We conducted partitioned Bayesian inference (BI) and maximum parsimony (MP) analyses based on the full concatenated and unphased dataset.

BI was carried out using MrBayes 3.1.2 [30]. The best-fit substitution models and partition were calculated using Partition Finder v1.1.1 [31], with Bayesian Information Criteria (BIC) suggesting the following scheme: K80+I for BDNF, HKY+I+G for CMOS, PDC and RAG2, GTR+I+G for 12S, 16S, and GTR+I+G for ND1. We performed two runs of 20 million generations (started on random trees) and four incrementally Markov chains (one cold and three heated, default heating values) each, sampling the Markov chains at intervals of 1000 generations. The convergence of parameter estimation across independent chains was visually checked with Tracer v1.5 [32] and mixing of chains was assessed with AWTY [33]. The first five million generations were conservatively discarded and 15,000 trees were retained post burn-in and summed to generate a 50%-majority rule consensus tree. Two independent BI analyses were also carried out on the concatenated mtDNA data set (12S, 16S and ND1) and on the unphased concatenated nDNA data set (BDNF, RAG2, CMOS, PDC), considering that congruence among mtDNA and nuclear DNA topologies represents an additional criterion supporting clade reliability [34].

MP analyses were performed using PAUP* 4.0b10 [35] with 100 random addition sequence replicates, equal character weighting, tree bisection and reconnection (TBR) branch swapping, and gaps coded as missing data. Nodal support was obtained by bootstrapping, with 2000 replicates, 10 random addition sequence replicates and TBR branch swapping.

We used two hierarchical outgroups: *Tiliqua* and ‘‘*Eumeces*” *sensu lato*. Among the non-Malagasy skinks, previous more inclusive studies [22], [36] suggested that species of the genus *Eumeces sensu lato* are relatively close to the Malagasy radiation. For both outgroup taxa, concatenated chimera datasets were compiled by combining sequences of different species from GenBank (see Crottini *et al*. [1]).

#### Time tree reconstruction

To estimate divergence times within the Malagasy Scincinae, we constructed a time calibrated phylogenetic tree using BEAST 1.8 package [37]. The fossil record of Malagasy crown reptiles is virtually unstudied, and under such circumstances the use of alternative calibrations such as "secondary" ones derived from previous divergence estimates may be considered useful [38]. Correspondingly, we calibrated the tree using node estimates and substitution rate estimates available in the literature. We retrieved the divergence time between the genus *Madascincus* and *Amphiglossus* (47 Ma; crown age of Malagasy scincines) from a comprehensive analysis of Malagasy vertebrate clades by Crottini *et al*. [39] based on multiple cross-validated primary fossil calibrations. The substitution rate prior was retrieved from a substitution rate study of Eo & DeWoody [40], which analysed mitochondrial substitution rates across several families of reptiles and birds and estimated a mean mitochondrial rate for squamates of 5.29 x 10^–9^ per site per year. To cross-validate these two priors we carried out two independent preliminary analyses: (i) we fixed the mitochondrial substitution rate at 0.0053/site/MYr and estimated divergence times both on the mtDNA dataset and on the whole dataset (mtDNA+nDNA) to see if the estimated median divergence between *Madascincus* and *Amphiglossus* would fall within the confidence interval reported by Crottini *et al*. [39]; ii) similarly, we fixed divergence between *Madascincu*s and *Amphiglossus* at 47 Ma and estimated the whole dataset substitution rate in order to evaluate if it approximated the one reported by Eo & DeWoody [40]. As both analyses showed that the two priors were compatible, iii) we proceeded with the time tree estimation on the whole dataset using the two prior values as calibrations. We used a normal prior for the divergence between *Madascincus* and *Amphiglossus* setting the mean at 47 Ma and the standard deviation (S.D.) at 12 MYr in accordance to Crottini *et al*. [39], and another normal prior for the mitochondrial substitution rate (mean: 0.0053/site/MYr, S.D.: 0.003, quantiles 5% = 3.6E-4, 95% = 0.01) in accordance to Eo & DeWoody [40]. Although these calibrations are not ideal (i.e., not primary fossil-based [38]), they provide a first objective approximation of divergence times of Malagasy skinks to be refined in future studies.

The best-fit substitution models and partition were calculated using Partition Finder v1.1.1 [31] (see above). Consequently, for the molecular clock model we divided the dataset into two partitions, one for the mitochondrial and another for the nuclear data, both with a relaxed molecular clock model with lognormal distribution [41]. Hence, the complete scheme consisted of three partitions for the substitution models and two partitions for the molecular clock model so that the substitution rates represent the mtDNA average and the nuclear DNA average. We used a Yule speciation tree prior, keeping only one sample per species. The MCMC chain was run for 5.0 x 10^7^ iterations sampled every 5.0 x 10^3^ steps; Three independent runs with different seeds yielded virtually identical results in topology, support and time estimates.

Chain mixing, Effective Sample Size (ESS) and convergence were visually accessed using Tracer v1.5 [32]. Exploratory analysis using the substitution model settings described above showed poor chain mixing and unacceptable ESS values below 200. The use of over-parameterized models for a given data set can cause poor chain mixing due to difficulty of parameter estimation since the information content of the data set may be insufficient for estimating all parameters of complex models. Thus, in an attempt to reach mixing and acceptable ESS we simplified the substitution models for the two mitochondrial partitions (we arbitrarily set both to HKY+I+G), and excluded two species (*Pseudoacontias menamainty* and *Amphiglossus crenni*) that had missing data for all nuclear genes. As a result, the simplified analysis showed good mixing and ESS values larger than 700, reaching stationarity in the beginning of the burn-in (first 10% of all iterations). We summarized a maximum credibility clade tree using Tree Annotator from Beast v1.8 package [37], discarding 10% as burn-in. The tree was visualized and edited using Figtree v1.4.1 [42].

#### Population genetic analyses

Population genetic structure was assessed within the clade containing *Grandidierina fierinensis* and individuals assigned by Glaw & Vences [19] to an undescribed candidate species named *G*. sp. “*pallida*” on the basis of their morphological differentiation. The PHASE algorithm [43] implemented in DnaSP v5 [44] was used to infer haplotypes from each available sequence of each nuclear gene, in a data set containing all *Grandidierina* species. Haplotype networks were reconstructed using statistical parsimony [45], as implemented in the program TCS v1.21 [46] with a connection limit of 95%. Analyses of population structure within the *G*. *fierinensis* clade were based on the four nuclear loci using STRUCTURE v2.2 [47] under a model assuming two populations (K = 2) in order to verify if the genetic data support differentiation of the two phenotypic groups found. This analysis assigns individuals probabilistically to clusters based on their multilocus genotype. We performed five replicates with two million MCMC generations of which 50% were discarded as burnin. We used a model that considers the possibility of mixed population ancestry and of correlated allele frequencies among populations due to migration or shared ancestry [47].

#### Analyses of character evolution

The evolution of two morphological characters (the degree of forelimb and hindlimb development) was traced along the tree topology obtained from the Bayesian analysis, using MP with PAUP* 4.0b10 [35]. Four different states were defined, separately for hind- and forelimbs: (0) *well-developed* (= presence of penta-, tetra- or tridactyl fore/hind-limbs), (I) *moderately regressed* (= presence of didactyl fore/hind-limbs), (II) *highly regressed* (= presence of monodactyl fore/hind-limbs or a digit-less limb-bud) and (III) *absent* (= absence of external fore/hind-limbs). As with any such analyses, a fully objective definition of character states can be challenging. A higher number of character states (i.e., defining loss of each digit as a separate state) is difficult for parsimony methods as used here which might detract from the putatively more relevant transformations (i.e., the full regression of limbs). Hence, because many non-fossorial skinks have 5, 4 or 3 digits we merged these variations into a "well-developed state" and thereby emphasized more strongly the transformations into the fully regressed states which we assume occur under selective pressure penalizing quadrupedal locomotion. Furthermore, we selected this scheme of character states because it can be consistently applied in the same way to the reductions seen in the forelimbs as well as to those seen in the hindlimbs observed in the various groups.

We used three character transformation options, defining all characters as either (i) unordered, i.e. defined such that any state is capable of transforming directly to any other state; (ii) ordered, i.e. defined such that the different states follow a linear and progressive succession of transformations, and (iii) according to the Camin-Sokal optimization, equivalent to ordered but with the additional constraint of irreversibility being imposed. For each of these three approaches, analyses were performed using ACCTRAN (accelerated transformations; favouring reversal over parallelism), and (ii) DELTRAN (delayed transformations; favouring parallelism over reversal). Formalization of the character states and character matrices are detailed in S1 Appendix.

## Results

### Phylogenetic relationships

**Fig 2:**
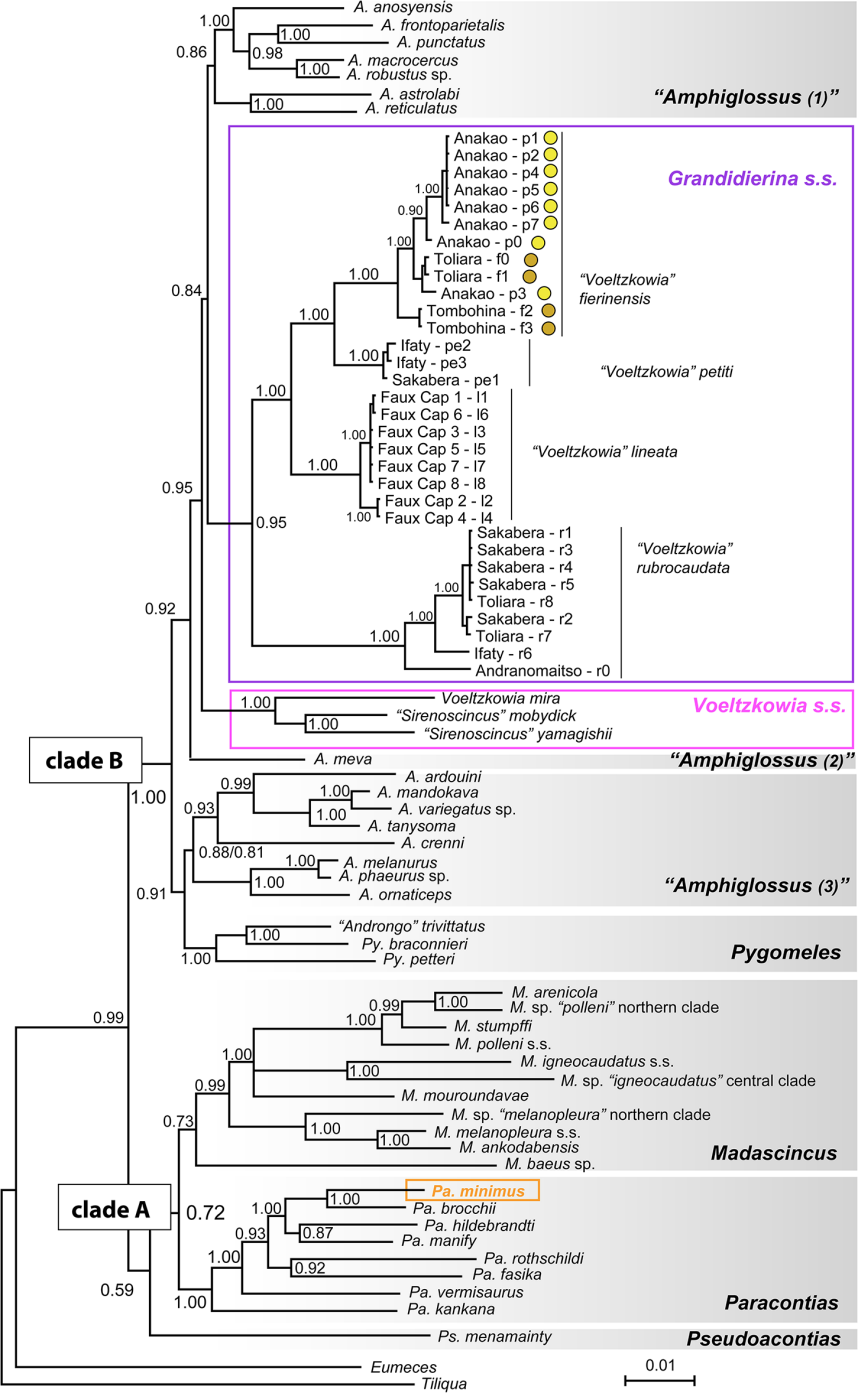
Phylogenetic tree of Malagasy scincines. Tree reconstructed using Bayesian Inference, based on concatenated DNA sequences of three mitochondrial (12S, 16S rRNA, ND1) and four nuclear (BDNF, C-mos, Rag2 and phosducin) loci, with posterior probabilities followed by bootstrap support values >50% from a Maximum Parsimony analysis. The three convergent clades including species with regressed eyes of the sand-swimming ecomorph are highlighted by coloured rectangles (note that *Pygomeles braconnieri* and *P*. *petteri* might also be considered as large-sized sand-swimmers). Species are shown with their previous genus-level classification (in quotation marks) while the larger font on the right side shows the revised classification. Within *G*. *fierinensis*, pale and dark phenotypes are indicated by yellow and brown circles, respectively.

The BI tree based on the complete concatenated dataset is highly congruent with previously published phylogenies [1], [7], [26]. It retrieves the two main clades A and B (cf. [36]) within Malagasy scincines (Fig 2). The poorly supported clade A is formed by the two monophyletic sister genera *Paracontias* (Posterior probability (PP): 1.00) and *Madascincus* (PP: 0.73), which together are sister to *Pseudoacontias menamainty*, a taxon of unstable relationships (but probably belonging to clade A) for which sequences of only 3 out of 7 loci are available. Clade B (PP: 1.00) is composed of the paraphyletic genus *Amphiglossus*, the *Grandidierina* clade (PP: 0.95), the *Pygomeles* clade (PP: 1.00), and the *Voeltzkowia* clade (1.00) (see taxonomic accounts in Discussion). Within *Pygomeles*, the species *Py*. *trivittatus* (formerly in a monotypic genus *Androngo*) was nested and placed sister to *Py*. *braconnieri* (PP: 1.00). Species of the sand-swimmer ecomorph were placed at three different positions in the tree: (i) *Paracontias minimus* was deeply nested within the genus *Paracontias*, (ii) the genus *Grandidierina* (comprising all the species formerly included in *Voeltzkowia* occurring in the south of Madagascar), and (iii) the genus *Voeltzkowia* containing the type species of this genus (*V*. *mira*) and the two species formerly included in *Sirenoscincus* (*V*. *mobydick*, *V*. *yamagishii*). Separate BI analyses of the nuclear and mitochondrial datasets (S2 Appendix) show overall similar topologies although many of the deeper nodes received lower support values. Both trees agree with the existence of three distinct lineages of small-sized sand-swimmers. The MP tree based on the complete dataset is relatively poorly resolved (CI = 0.34, HI = 0.66, RI = 0.63) but does not present relevant incongruences compared to the BI tree (S2 Appendix). It retrieves the monophyly of clade A (bootstrap score = 86%), and of the *Grandidierina* (84%), *Paracontias* (56%), *Pygomeles* (60%) and *Voeltzkowia* (99%) clades, with the same content and internal species relationships as suggested by BI.

### Comparative anatomy of the appendicular skeleton

**Fig 3:**
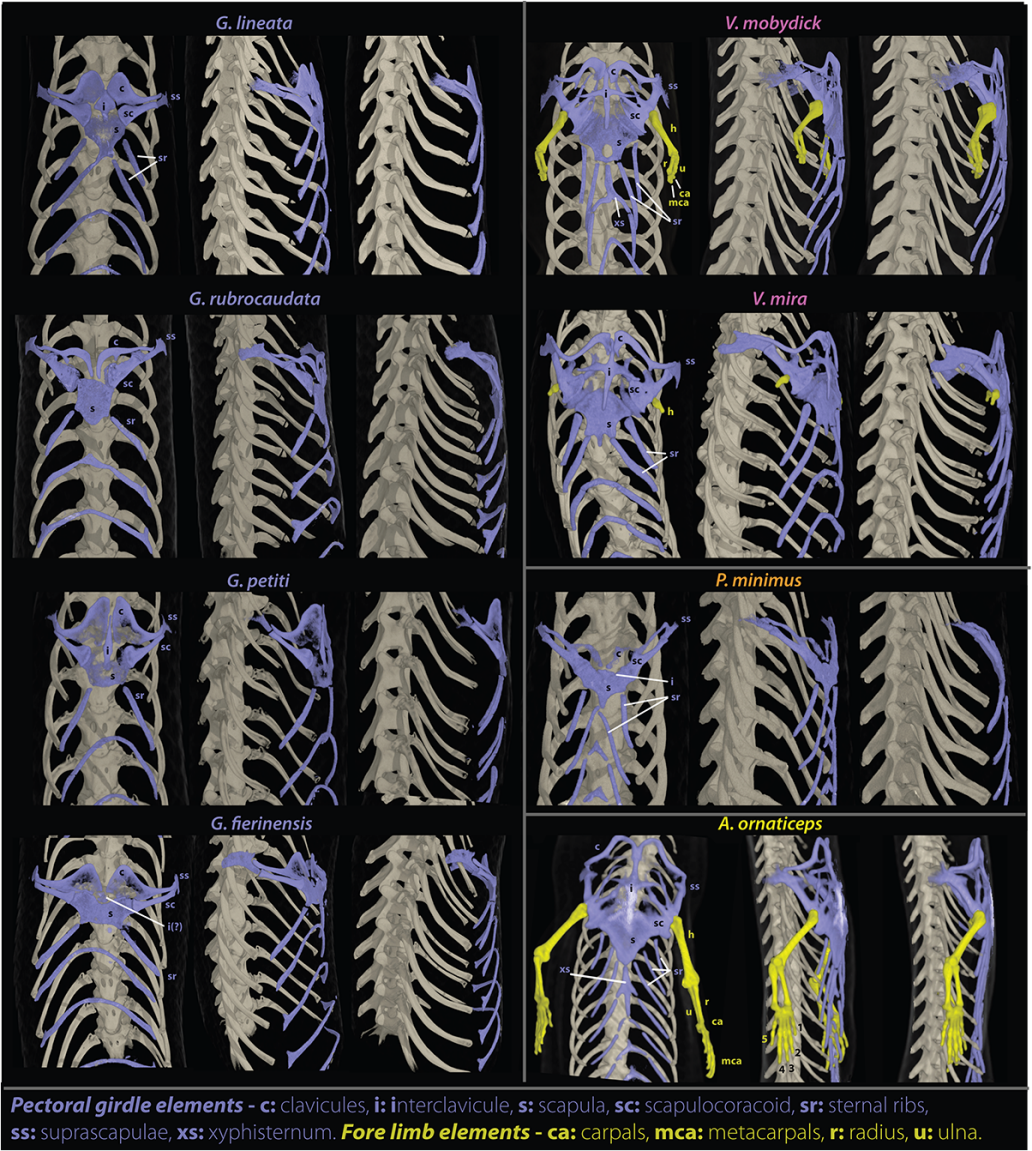
Pectoral girdle and forelimbs skeletal anatomy. Computed tomographic reconstruction of the pectoral girdle (in purple) and forelimbs (in yellow) of seven different species of blind sand-swimming skinks and one species without limb regression (*Amphiglossus ornaticeps*), based on microtomographic scans, in ventral, ventrolateral and lateral view (not to scale).

**Fig 4:**
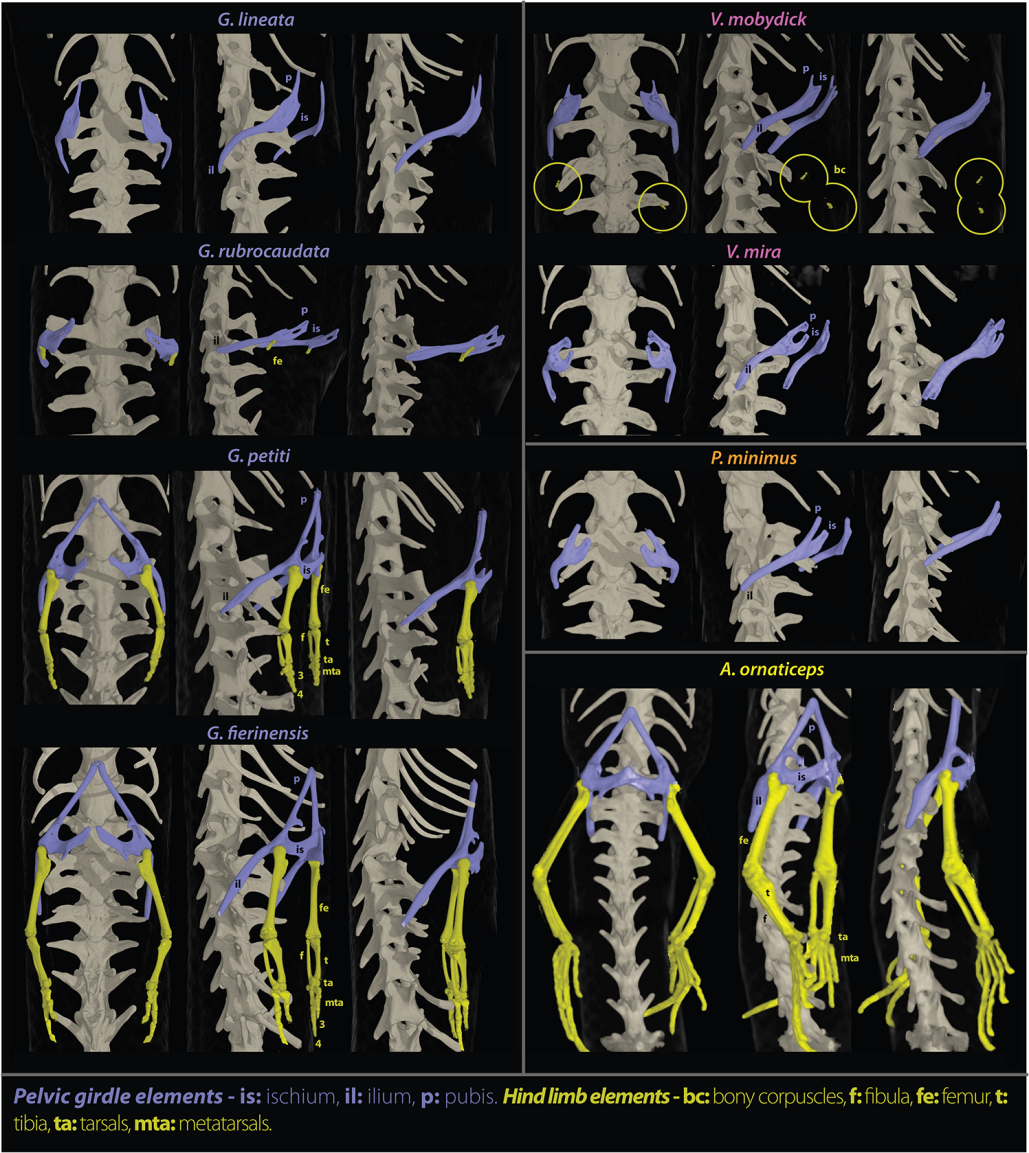
Pelvic girdle and forelimbs skeletal anatomy. Computed tomographic reconstruction of the pelvic girdle (in purple) and hindlimbs (in yellow) of seven different species of blind sand-swimming skinks and one species without limb regression (*Amphiglossus ornaticeps*), based on microtomographic scans, in ventral, ventrolateral and lateral view (not to scale).

*Amphiglossus ornaticeps* presents well developed pectoral and pelvic girdles with complete pentadactyl and functional autopods, considered here as the typical plesiomorphic state expected in fully quadrupedal skinks [24], [48],[49]. In contrast, the examined limbless or bipedal species of the genera *Grandidierina*, *Voeltzkowia* and *Paracontias* show different degree of regression of girdles and autopods (Figs 3 and 4) (cf. S1 Text for detailed descriptions of each species' appendicular anatomy):

In the legless or postero-bipedal species of the genus *Grandidierina*, forelimbs have completely disappeared with no traces of relictual autopodial bones. The pectoral girdle is structurally relatively regressed but highly ossified, tending to form a compact “pectoral shield” relatively short in length, with the interclavicula being either very reduced or absent. Hindlimbs are significantly regressed but present a variety of degree of reduction: the plesiomorphic autopodial structure is relatively well preserved in postero-bipedal species (in the two-toed *G*. *fierinensis* or single-toed *G*. *petiti*), extremely regressed in the legless species (in *G*. *rubrocaudata* consisting of only a pair of extremely reduced pear-shaped femurs articulating with the pelvis through a relatively well developed acetabular fossa), or completely regressed with no vestigial bones (legless *G*. *lineata*). The pelvic girdles are relatively developed in both postero-bipedal species, and the hemipelves conserve a typical trifurcate aspect, whereas they are highly reduced in the two legless species (the pelvis and the ischium tending to fuse together, conferring a curved and rod-like aspect to each hemipelvis).

In the legless or antero-bipedal genus *Voeltzkowia*, forelimbs are significantly regressed with a variety of degree of reduction: the typical plesiomorphic autopodial structure is still recognizable (in the fingerless antero-bipedal *V*. *mobydick*, relictual carpals and metacarpals remain but are hardly identifiable) or extremely regressed but not completely reduced (in the legless *V*. *mira* subsisting only a pair of extremely reduced pear-shaped humeri articulating with the scapulocoracoid through a relatively well developed glenoid fossa). The pectoral girdle is well preserved, relatively similar to the one of the fully quadrupedal *A*. *ornaticeps*, although slightly more ossified and compact. Hindlimbs are completely regressed in the legless *V*. *mira*, whereas two hardly distinguishable bony corpuscles, probably representing rudiments of ancestral hindlimb bones, are still present in the anterobipedal *V*. *mobydick*. The pelvic girdle is highly regressed, the hemipelves being relatively elongated in a rod-like structure, although the distal extremities of the pelvis and ischium are still well differentiated in *V*. *mira*.

In the strictly legless genus *Paracontias* (only *P*. *minimus* examined herein), both forelimbs and hindlimbs are completely regressed, with no vestigial bones. The pectoral girdle is extremely regressed, two times wider than long, tending to form a chevron-shaped pectoral shield. The pelvic girdle is highly regressed, the hemipelvis being relatively elongated in a rod-like structure, although the distal extremities of the pelvis and ischium are still well differentiated.

### Parallelism and reversal in limb regression characters

**Fig 5:**
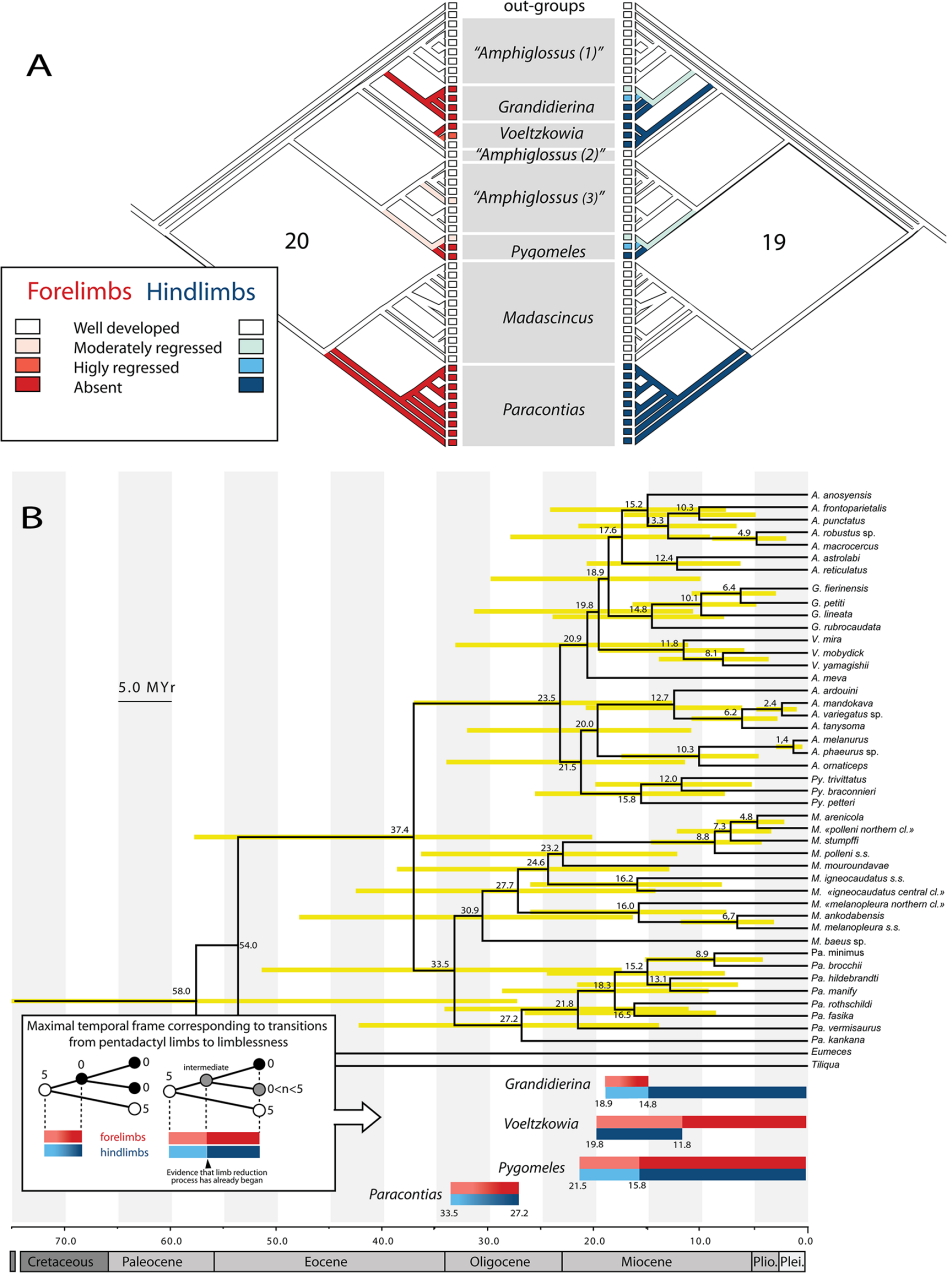
Evolution of limb regression over time. (A) Evolutionary regression of forelimbs and hindlimbs within the Malagasy scincine lineage. Character evolution is reconstructed using three parsimony optimizations implemented in PAUP 4.0b10 with an ordered and irreversible algorithm (Camin-Sokal parsimony). Character evolution is represented on the left side for the forelimbs (red gradient), and on the right side for the hindlimbs (blue gradient). Numbers represent the number of character changes involved in each reconstruction. (B) Time tree of Malagasy scincines, using a Bayesian relaxed-clock phylogenetic approach, with two normal priors used for time calibration: the divergence between *Madascincus* and *Amphiglossus* 47 Mya, as retrieved from a previous more comprehensive analysis of Malagasy vertebrates [8], and a mitochondrial substitution rate of 0.0053/site/MYr (see methods for details).Yellow bars at nodes indicate 95% credibility intervals for divergence events. Temporal scale is shown in millions of years. Blue and red bars are maximal temporal windows for limb regression for the four limbless lineages presented in the tree, based on the ancestral state reconstruction with ordered and irreversible characters, and delimited by the last node fitting with a fully pentadactyl inferred ancestor and the first node fitting with an inferred ancestor with no external limbs. Intervals are shown for forelimbs with red bars, and for hindlimbs with blue bars. Where applicable, the maximal temporal frame for initiation of the limb regression process (delimited by the last node fitting with a fully pentadactyl ancestor and the first node fitting with an ancestor with less than five fingers) is represented by light red / blue colors.

The MP approaches to ancestral state reconstruction (Fig 5A, see also S2 Appendix) reconstructed 8 and 9 evolutionary steps (forelimbs and hindlimbs, respectively) with unordered characters, 20 and 18 steps with ordered characters, and 20 and 19 steps with irreversible characters. The two approaches, i.e. assuming unordered and ordered character states, respectively, present obvious and expected differences, but both suggest the occurrence of reversals: 2 and 5 for the forelimbs and 4 and 7 for the hindlimbs (ACCTRAN); 1 and 0 reversals for the forelimbs, and 2 and 1 for the hindlimbs (DELTRAN).

The assumption of irreversible character states leads to a hypothesis of five clades, i.e., *Pseudoacontias*, *Paracontias*, *Pygomeles*, *Grandidierina* and *Voeltzkowia*, having independently evolved toward snake-like forms from fully quadrupedal and pentadactyl ancestors. Also, in this reconstruction, within *Pygomeles*, *Grandidierina* and *Voeltzkowia*, several species have lost limbs (postero- or antero-bipedalism or complete leglessness) several times independently. A very similar reconstruction was obtained with ordered character states and delayed transformation that differed only by a single, relatively minor, reversal in *Grandidierina* (re-evolution of a digit in the two-toed *G*. *fierinensis* from a single-toed ancestor). Considering empirical evidence from other squamates and vertebrates (see Discussion) we regard such a no-reversal hypothesis to be reasonable in Malagasy skinks and applied it in subsequent analyses (Fig 5A).

### Tempo of limb regression in Malagasy skinks

Time trees calculated from the two independent preliminary analyses (see approaches *i* and *ii* described in subsection *Time tree reconstruction*in Methods) showed comparable results with confidence intervals of estimates largely overlapping. When using a fixed prior for the divergence of *Madascincus* and *Amphiglossus*, the resulting mitochondrial substitution rate (0.0051/site/MYr, 95% HPD: 0.0040–0.0063) is close to the rate reported for squamates (see Eo & DeWoody [40]. When a fixed substitution rate is used (0.0053/site/MYr), the resulting divergence between *Madascincus* and *Amphiglossus* is 37.4 MYr (95% HPD: 35–53Mya) with a confidence interval falling within the one reported by Crottini *et al*. [39]. These results show that both priors are compatible (cf. S3 Appendix). Thus, we favoured the analysis using two normal priors as it contains more prior information and we discuss only this result (Fig 5B). The evolutionary time needed for limb regression has been inferred based on the preferred age estimates for the nodes and the no-reversal ancestral state reconstruction. This approach suggests that loss of both fore- and hindlimbs, i.e., the transition from the last fully quadrupedal and pentadactyl inferred ancestor to the first inferred completely legless ancestor, would have taken place in *Paracontias* 33.5 to 27.2 Ma, complete loss of forelimbs in *Grandidierina* from 18.9 to 14.8 Ma, and complete loss of hindlimbs in *Voeltzkowia* from 19.8 to 11.8 Ma. Regression of forelimbs and hindlimbs in *Pygomeles* started between 21.5 and 15.8 Ma. Regression of hindlimbs in *Grandidierina*, and of forelimbs in *Voeltzkowia*, started between 18.9 and 14.8 Ma, and between 19.8 and 11.8 Ma, respectively.

### Phenotypic and genetic differentiation within *Grandidierina fierinensis*

**Fig 6:**
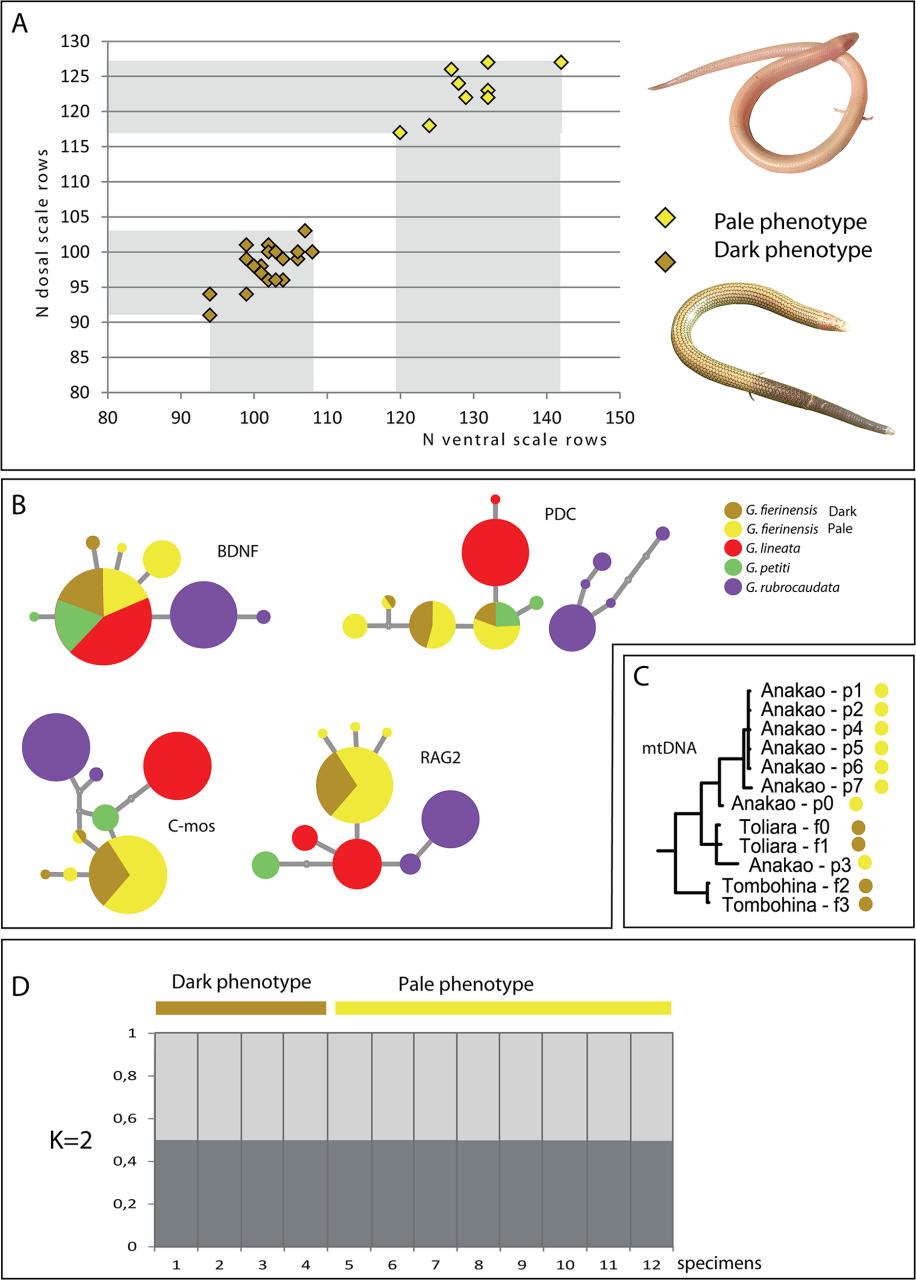
Morphological versus molecular differentiation between the dark and pale phenotype of *G*. *fierinensis*. (A) Number of ventral and dorsal scales along the body. (B) Haplotype network reconstruction including all the species of *Grandidierina*. Note the elevated amount of shared haplotypes among the two *G*. *fierinensis* phenotypes. (C) Mitochondrial divergence within the *G*. *fierinensis* complex (subtree extracted from the result of the Bayesian analysis carried out on the concatenated mtDNA sequences, cf. S2 Appendix). (D) Genetic structure inferred from four nuclear loci across 12 individuals of *G*. *fierinensis*, with K = 2; assignment probability of each specimen (N = 12) is around 50% for either cluster, independent of its phenotype.

All *Grandidierina fierinensis* specimens could be unambiguously assigned to either of two well differentiated colour morphs (dark and pale), with apparently no intermediate state. Both forms share the same background colouration in preservative, ventrally whitish and pale yellow dorsally. The dark phenotype, from Toliara, Tombohina and Betioky, is characterized by the presence of a dark pigmented margin highlighting the posterior border of each cycloid scale, therefore conferring a grayish overall aspect. The pale phenotype, from Anakao (corresponding to *G*. sp. “pallida” of Glaw & Vences [19], has unpigmented scale margins (Fig 6A) and is larger than the pigmented phenotype (mean of the snout-vent length = 75.6 mm ±18.2, N = 11; versus 61.1 mm ± 10.6, N = 26). The two phenotypes also differ substantially in various scale counts: Pale specimens have more ventrals (120–142, mean = 129.6 ± 6.2, N = 9; versus 94–108, 101.7 ± 3.7; N = 26) and dorsals (117–127, 122.3 ± 3.9, N = 10; versus 91–103, 97.6 ± 3.22, N = 27), without overlapping values (Fig 6A).

Haplotype networks inferred from the phased nuclear DNA sequences of *Grandidierina* show a limited amount of haplotypes shared among the four described species of the genus (*G*. *fierinensis*, *G*. *lineata*, *G*. *petiti* and *G*. *rubrocaudata*): none in C-mos and RAG2 and one each in PDC and BDNF, between *G*. *fierinensis* and *G*. *petiti*, and between *G*. *fierinensis*, *G*. *lineata* and *G*. *petiti*, respectively (Fig 6B). The central position of these unique shared copies within the network and their elevated frequency of occurrence (especially in BDNF) suggest ancestral haplotype sharing although recent gene flow obviously cannot be excluded. The dark and pale phenotypes of *G*. *fierinensis* do not show any obvious pattern of genetic differentiation and share haplotypes of the four nuclear markers analysed. Neither the tree of the concatenated mitochondrial and nuclear data (Fig 2) nor the tree based on mtDNA only (Fig 6C and S2 Appendix) resolves pale or dark specimens as monophyletic, although seven out of the eight analysed samples of the pale form are grouped in a shallow clade. Population structure analysis of nuclear loci with K = 2 results in all specimens having equal assignment probability for the two clusters (Fig 6D). Although it is not possible to reliably date the morphological changes within *G*. *fierinensis* due to the lack of reciprocal monophyly of the two morphologies, it is expected that such phenotypic differentiation took place at a significantly younger date than the separation between *G*. *fierinensis* and *G*. *petiti* (6.4 Ma according to the two-prior divergence time analysis).

## Discussion

### Revised taxonomic classification of *Voeltzkowia*, *Grandidierina*, and *Pygomeles*

The tree topology obtained in the present study reveals the inadequacies in the current definition of several genera of Malagasy scincines. While the need for further taxonomic rearrangement was already recognized in previous studies [1], the inclusion of several crucial taxa herein allows formalization of some taxonomic conclusions. In particular, our molecular phylogeny is the first to include *V*. *mira* (type species of *Voeltzkowia*), *Pygomeles petteri* (the second species of *Pygomeles*), and the two species described in the genus *Sirenoscincus*. Our data suggest (i) that the south-western species previously considered as *Voeltzkowia* form a distinct clade in which *V*. *mira* is not included, (ii) *V*. *mira* forms a clade with the two species currently included in *Sirenoscincus*, and (iii) *Pygomeles* is paraphyletic due to the inclusion of one species (*trivittatus*) currently considered as the sole species of *Androngo* (see discussion in Crottini *et al*. [1]). Based on these results, we propose the following genus-level rearrangements, and for each of these we explain our rationale based on explicit Taxon Naming Criteria (TNCs) [50]:

#### Voeltzkowia Boettger, 1893

This genus currently contains five species [21]. We here propose a revised classification including the following three species: (1) *V*. *mira* Boettger, 1893; (2) *V*. *yamagishii* (Sakata & Hikida, 2003), *comb*. *nov*.; and (3) *V*. *mobydick* (Miralles, Anjeriniaina, Hipsley, Müller, Glaw & Vences, 2012), *comb*. *nov*. *Voeltzkowia mira* being the type species of the genus, we propose to apply this generic name to all the species of the north-western clade. All of these are characterized by hindlimb regression prevailing on those of the forelimbs (relictual presence of forelimb bones only, and a pectoral girdle well-developed resembling those usually present in quadrupedal skinks, even in the completely legless *V*. *mira*). This implies considering the name *Sirenoscincus* Sakata & Hikida, 2003 as a junior synonym of *Voeltzkowia*. This solution satisfies two of the three primary TNCs (monophyly, clade stability) and partially the third one (diagnosibility) albeit only based on internal characters. It also satisfies the Biogeography TNC because all three species occur in a well-defined geographical area. The alternative solution (a monotypic *Voeltzkowia* with only *V*. *mira*, and a separate monophyletic genus *Sirenoscincus* with two species) would also yield a monophyletic and stable unit and an externally diagnosable *Sirenoscincus* (based on forelimb rudiments). This solution is however not favoured because (i) the morphological differences between the three species are gradual, and (ii) the genetic distances (see branch lengths in Fig 2) between these three species are relatively low, similar to those within other genera such as *Paracontias* or *Pygomeles*. Therefore, grouping them in the same genus agrees better with the time banding TNC [50].

#### Grandidierina Mocquard, 1894

This genus has been initially intended to include two species originally described under the names *Acontias rubrocaudatus* and *Scelotes fierinensis*. It has been subsequently downgraded to a subgenus of *Voeltzkowia* to accommodate the two species with reduced hindlimbs (*V*. *fierinensis* and *V*. *petiti*) whereas the entirely limbless species (*V*. *mira*, *V*. *lineata* and *V*. *rubrocaudata*) were placed in the subgenus *Voeltzkowia* [21]. *Scelotes fierinensis* having been designated as the type species of the genus *Grandidierina* by Brygoo [21], this generic name is here resurrected and applied to all the species of the south-western clade, which are anatomically characterized by forelimb regression prevailing over hindlimb regression: when limb bones are present, relictual bones are only those of hindlimbs, and the pectoral girdle is relatively regressed, with a scapulacoracoid very regressed and elongated, flattened clavicles, and interclavicles either very regressed or absent. We recognize four described species belonging to the genus *Grandidierina*, which are: (1) *G*. *fierinensis* (Grandidier, 1869); (2) *G*. *rubrocaudata* (Grandidier, 1869); (3) *G*. *lineata*, Mocquard, 1901; and (4) *G*. *petiti* Angel, 1924.

#### Pygomeles Grandidier, 1867

This genus currently contains two species, the type species *Py*. *braconnieri* and the subsequently described *Py*. *petteri* [51]. The phylogeny of Crottini *et al*. [1] included only *Py*. *braconnieri*, a species from south-western Madagascar. This species was found to be the closely related sister group of another southern species with reduced limbs classified as *Androngo trivittatus*. However, because *Py*. *petteri* was missing from their data set, Crottini *et al*. [1] refrained from taxonomic conclusions. Our analysis (Fig 2) suggests that *An*. *trivittatus* is the sister taxon of *Py*. *braconnieri* and these two taxa form the sister clade *to Py*. *petteri*, thus rendering *Pygomeles* sensu lato paraphyletic. Given that also the genetic distances among these three species are not larger than within other skink genera (see branch lengths in Fig 2), we propose to include *trivittatus* in the genus *Pygomeles* to obtain a classification satisfying the monophyly and clade stability TNCs. Because *trivittatus* is the type species of the genus *Androngo* this implies considering *Androngo* Brygoo, 1982 as junior synonym of *Pygomeles*. The genus *Pygomeles* thus includes the three species: (1) *Py*. *braconnieri* Grandidier, 1867; (2) *Py*. *petteri* Pasteur & Paulian, 1962; and (3) *Py*. *trivittatus* Boulenger, 1896 plus the subspecies *Py*. *trivittatus trilineatus* (Angel, 1949), which is morphologically distinguishable by larger size, a complete number of five fingers and toes, and a higher number of presacral vertebrae [52], but has not yet been studied genetically.

These rearrangements leave only the paraphyletic genus *Amphiglossus* in need of a revised classification which we will undertake in a forthcoming study including additional markers and a wider taxon sampling of this genus.

### Parallel limb regression in Malagasy skinks

In agreement with previous studies [1], [22], [23], our data support multiple origins of limb regression in Malagasy skinks. According to the new taxonomy proposed here, limb regression is found in five different genera, each of which also includes at least one entirely limbless species: *Pseudoacontias*, *Paracontias*, *Pygomeles*, *Grandidierina*, and *Voeltzkowia*. Of these, *Grandidierina*, *Voeltzkowia*, and one species of *Paracontias* clearly are sand-swimmers, and the larger-sized *Pygomeles braconnieri* and *P*. *petteri* can probably also be assigned to this ecomorph.

Due to poor support for several basal nodes in our tree we cannot exclude that within clade A, *Pseudoacontias* and *Paracontias* might be sister genera, and within clade B, either combination of *Grandidierina*, *Pygomeles*, or *Voeltzkowia* might form a monophyletic group. However, each of these genera also contains taxa with limb rudiments, and we do not favour reconstructions of fully limbless ancestors for the respective clades. Such reconstructions (as e.g. suggested if characters are defined as unordered and accelerated character state transformation selected) imply a substantial number of reversals, i.e., re-evolution of lost autopodial structures. Even the most conservative approach, ordered characters with delayed transformations, still reconstructed one such reversal. However, empirical evidence from vertebrate paleontology, developmental biology and phylogenetics (e.g., in archosaurs [53], cetaceans [54], snakes [55] or other lizards [56]) supports as more realistic assumption of gradual transformation, with few or no reversals.

Although mutations affecting single genes such as Tbx5 or WNT3 are known to involve dramatic developmental disruptions resulting in sudden limb loss in quadrupedal vertebrates [57], [58], they are usually associated with strong detrimental pleiotropic effects, and we therefore hypothesize that such dramatic and sudden mutations only exceptionally, if at all, influence vertebrate evolution. Among the plethora of papers published on limb loss in lizards, a majority supports the irreversibility statement as well [11], [23], [59]. Only a few studies [16], [60], [61] indicated possible reversals, but usually affecting minor transformations such as secondary gain of single digits, at low frequencies, or based on disputed reconstructions [62], [63]. Therefore we here rely on the reconstruction assuming irreversible transformations that supports multiple parallel processes towards limblessness in Malagasy scincines.

Further evidence for such parallel evolution comes from the different pattern observed in *Grandidierina* vs. *Voeltzkowia* and *Pygomeles*, with regression of forelimbs preceding that of hindlimbs or vice-versa. In fact, by recovering a highly supported *Voeltzkowia* clade with *V*. *mira*, *V*. *mobydick* and *V*. *yamagishii*, we provide for the first time conclusive evidence that a hindlimbs-first regression pattern can also lead to fully limbless species such as *V*. *mira*, with rudiments of forelimb bones still present but hindlimb bones completely regressed. As stressed by Miralles *et al*. [8] this hindlimbs-first regression pattern is exceptional among squamates while a more pronounced regression of forelimbs is typically encountered [16]. Consequently, postero-bipedal species (presence of hind-limbs only) are much more frequently observed within limb regressed squamates, and antero-bipedal species (presence of forelimbs only) are only known from *Voeltzkowia* (formerly *Sirenoscincus*), and from the amphisbaenian *Bipes* and the Asian skink *Jarujinia* [6], [8], [15], [64].

### Temporal patterns of limb regression

Despite uncertainties in the absolute time estimates, our data allow several inferences on the relative chronology of limb regression in Malagasy skinks. It is likely that completion of the Early Oligocene process of limb regression in *Paracontias* preceded the start of limb regression in the other three clades (*Pseudoacontias* not considered here due to its unresolved phylogenetic placement). The only species in the genus examined morphologically herein, *Paracontias minimus*, has pelvic and especially pectoral girdles more regressed than *Voeltzkowia* and *Grandidierina*, suggesting that girdle elements might continue to regress gradually with time even after limbs and autopodial bones have already disappeared. Within *Paracontias*, only the phylogenetically nested *P*. *minimus* is a sand swimmer with regressed eyes and, thus, limb regression in this genus might have taken place during a specialization to litter-burrowing habits within humid forests that expanded in Madagascar in the Eocene [65]. Limb regression in *Grandidierina*, *Voeltzkowia* and *Pygomeles* started rather concurrently, probably in the Early Miocene. No thorough bioclimatic reconstructions exist for Madagascar during this period, but the parallel evolution of three clades of skinks of the sand-swimming ecomorph in different coastal regions of Madagascar (southwest, northwest and north) might suggest emergence of new, dry and sandy ecosystems during this period.

Our analysis also suggests that important macroevolutionary transformations, such as complete regression of limbs, might happen in comparatively short time frames. In our analysis the maximal duration of the complete transition from a fully quadrupedal pentadactyl form to a limbless form did not exceed ≈ 6 MYr in the ancestors of *Paracontias*, and complete regression of hindlimbs (*Voeltzkowia*) or forelimbs (*Grandidierina*) required comparable time spans (≈ 8 and ≈ 4 MYr at maximum, respectively). Despite large age credibility intervals for most nodes (Fig 5), these transition times still provide a strong contrast to previous estimates of limb loss duration in reptiles. For example, in a study encompassing multiple taxa across squamates [16], Brandley *et al*. found time spans of 16–176 MYr for such regressions, but acknowledged that faster regressions might be found with denser taxon sampling. Our results agree with this hypothesis and, if confirmed in future studies, would provide one of the shortest transition times towards limblessness, along with *Lerista* skinks (3.6 MYr [11]). One of the most important challenges for future analyses will be to reduce the uncertainty in the divergence time estimates, by inclusion of a larger number of loci and internal calibrations. It needs to be stressed once more that our hypothetical time frame of limb regression posited here relies on the preferred time estimates but does not take into account the wide confidence intervals that characterize these.

### Morphological versus molecular differentiation in *Grandidierina fierinensis*

Glaw & Vences [19] defined a population of skinks from Anakao in south-western Madagascar as candidate species, *Voeltzkowia* sp. "pallida" (herein *Grandidierina* sp. "pallida"), mainly based on its largely unpigmented body, number of scale rows around midbody, and longitudinal count of ventral scales, all of which were different from the other two-toed species of *Grandidierina*, *G*. *fierinensis*. Moreover, Bill Love (personal communication) took pictures of a specimen from Itampalo (around 110 km south of Anakao) apparently related to *Grandidierina fierinensis* but presenting the same unpigmented pattern as specimens from Anakao, and living in a similar habitat consisting of coastal sand dunes. Herein we refer to these specimens as the pale phenotype and confirm its morphological distinctness from the dark phenotype which likely corresponds to typical *G*. *fierinensis*. The holotype of this taxon (MNHN 1895–214) from “Tuléar” (= Toliara) upon our examination was uniformly pale but the original description [66] mentions a pattern of "petites lunules noiratres" and its low number of 96 dorsal scale rows is consistent with values in other (dark) specimens collected in the vicinity of Toliara, suggesting its original pigmentation might have faded after 120 years in preservative.

Although the dark specimens from Toliara and Tombohina and the pale specimens from Anakao can clearly be diagnosed morphologically, they do not form reciprocally monophyletic groups nor unambiguous population genetic clusters, which indicates ongoing gene flow between them, or a very recent origin with incomplete lineage sorting. Although further study is needed to determine their taxonomic status, at present we feel that all these specimens should be tentatively considered as representing a single species, and the two phenotypes might be explained with strong selective pressures for local adaptation. To our knowledge, the pale phenotype inhabits coastal dunes of fine-grained whitish sand whereas the dark form is found more inland with darker and more heterogeneous sand, including organic particles and gravel. The color differences between the two forms may thus either be mimetic or thermoregulatory, the pale color limiting heat gain [67].

Although we did not assess possible osteological character variation between the pale vs. dark phenotype, the strong and diagnostic differences in scale counts are striking. The number of dorsal and ventral scale rows is a common taxonomic character in squamates, and such important differences in these variables usually correlate with strong and unambiguous genetic divergences. The strong and possibly intraspecific difference seen here can be taken as further evidence for strong selective pressures during the adaptation to sand swimming, with the more fine-grained and white coastal sand dunes probably leading to more elongated bodies with higher numbers of scale rows. Although we have not studied the sex of the pale and the darker specimens, we are confident to exclude sexual dimorphism as possible explanation of this phenomenon due to the strictly allopatric distribution of both phenotypes.

Very poor genetic differentiation has also been detected in other morphologically distinct Malagasy scincines, e. g. *A*. *mandokava* and *A*. sp.“*variegatus*” [1], in some Malagasy frogs of the genus *Scaphiophryne* [68], and in different morphs of the European cave dwelling olm *Proteus anguinus* [69], but this phenomenon remains poorly understood.

### A slippery slope toward limblessness?

Limb regression followed by limb loss is frequently encountered among tetrapods. The most dramatic transformations occurred in snakes and in caecilians where the four limbs and the associated pectoral and pelvic girdles have been completely lost [70]. Evolutionary transformation of a fully quadrupedal, lizard-like body form to an almost or completely legless, elongate, worm-like body form have also repeatedly occurred in several lineages of Squamata other than snakes, i.e., in Amphisbaenia, Scincidae, Anguidae, Dibamidae, Pygopodidae, Gymnophthalmidae and Cordylidae [8]. Other tetrapods lost either their hindlimbs when adapting to aquatic environments (cetaceans and sirenians [55], [71]), or their forelimbs in the context of becoming flightless (the New Zealand Moas [72]).

It is particularly appealing in squamates to hypothesize that taxa with fully regressed limbs might be more successful than intermediate forms, suggested by the large number of completely limbless species especially of snakes and amphisbaenians, with 3495 and 187 species, respectively [73]. A thorough test of this hypothesis using comparative methods is however lacking and will be a fruitful endeavor for future studies, making use of comprehensive phylogenies now available [74]. In general, limbless body forms are often associated with smaller geographical range size [75] and thus possibly with reduced dispersal ability, and higher speciation and extinction rates, but it is unlikely that this constitutes a relevant difference to the species with almost limbless intermediate phenotypes.

Among Malagasy skinks, all clades with bipedal body form (*Grandidierina*, *Voeltzkowia* and *Pygomeles*) also include completely limbless species. On the contrary, two clades contain fully limbless species only, without intermediate forms (*Paracontias* and *Pseudoacontias*) and have probably started the limb regression process considerably earlier. Intermediate forms (species with less than five digits but retaining at least an external relict of limb) according to our divergence time estimate might have persisted for ≈ 5 to 20 MYr (at least ≈ 12 MYr in *Voeltzkowia*, ≈ 15 MYr in *Grandidierina*, ≈ 16 MYr for *Pygomeles* and at most 6 MYr in *Paracontias*), in relative agreement with Brandley *et al*. [16] who also found evidence for long-time persistence of such intermediates, from 9–63 MYr (median 27 MYr). Still, counting all species of *Grandidierina*, *Paracontias*, *Pseudoacontias*, *Pygomeles*, and *Voeltzkowia*, the completely limbless species (N = 20) clearly outnumber the intermediate forms (N = 6). Taken together, this might indicate that only a limited number of niches exist for intermediate phenotypes, even if under particular conditions they can persist for long evolutionary times. In this scenario, completely legless forms would tend to replace the intermediate forms because of their better adaptation to the specialized burrowing niche, and this in turn would imply that even tiny, relictual limbs would negatively impact the fitness of the intermediates. Alternatively, the continued regression of such tiny rudiments might be explained by drift, genes involved in the development of non-essential limbs tending to accumulate more rapidly deleterious mutations, or by a combination of drift and selection as it has been argued for the loss of eyes in species of cave fishes [76].

## Supporting Information

S1 Appendix Ancestral state reconstructions. (DOC)

S2 Appendix Complementary phylogenetic analyses of Malagasy scincines. (DOC)

S3 Appendix Time trees of Malagasy scincines. (DOC)

S1 Table List of voucher specimens, Genbank accession numbers and localities. (DOC)

S1 Text Osteological descriptions. (DOC)
