# Supplementary material for: Distinct Patterns of Desynchronized Limb Regression in Malagasy Scincine Lizards (Squamata, Scincidae)
Source: PLoS One. 2015 Jun 4;10(6):e0126074. doi: 10.1371/journal.pone.0126074 (PMC4456255; doi:10.1371/journal.pone.0126074)
Supplement: S1 Appendix — (DOC) [file pone.0126074.s001.doc]

**S1 Appendix. Ancestral state reconstructions**

**S1A.** Formalization and algorithms tested. The evolution of two morphological characters (forelimbs and hindlimbs degree of development) was traced along the tree topology obtained from the Bayesian analysis, und MP in PAUP* 4.0b10 (Swofford, 2002). Four different states were defined, separately for hind- and forelimbs (a): (0) *well-developed* (= presence of penta-, tetra- or tridactyl fore/hind-limbs), (I) *moderately regressed* (= presence of didactyl fore/hind-limbs), (II) *highly regressed* (= presence of monodactyl fore/hind-limbs or a digit-less limb-bud) and (III) *absent* (= absence of external fore/hind-limbs). We used three character transformation options (b), defining all characters as either (i) unordered, i.e. defined such that any state is capable of transforming directly to any other state; (ii) ordered, i.e. defined such that the different states follow a linear and progressive succession of transformations, and (iii) according to the Camin-Sokal optimization, equivalent to ordered but with the additional constraint of irreversibility being imposed. For each of these three approaches, analyses were performed using ACCTRAN (accelerated transformations; favoring reversal over parallelism), and (ii) DELTRAN (delayed transformations; favoring parallelism over reversal). Different approaches of ancestral state reconstruction based on parsimony have been applied using PAUP* 4.0b10 (Swofford, 2002). Different optimization algorithms already implemented in the software have been tested: (1) the “Fitch optimization” where the characters are unordered, i.e. defined such that any state is capable of transforming directly to any other state (UNORD); (2) the “Wagner optimization” where characters are ordered, i.e. defined such that the different states follow a linear and progressive succession of transformations (ORD) and (3) the “Camin-Sokal optimization”, equivalent to Wagner parsimony with the additional constraint of irreversibility being imposed (IRREV). Noteworthly, the “Dollo optimization” (DOLLO), where characters are consistent with the requirement that every derived states are allowed to originate only once during evolution (all homoplasy taking the form of reversals to a more ancestral state) has been tested although such prerequisites does not make biological sense in the present context (homoplasy between tetrapodal species would always be explained by re-evolution of limbs/digits, the convergent loss between different legless species being not allowed), and has therefore not been included in this study.


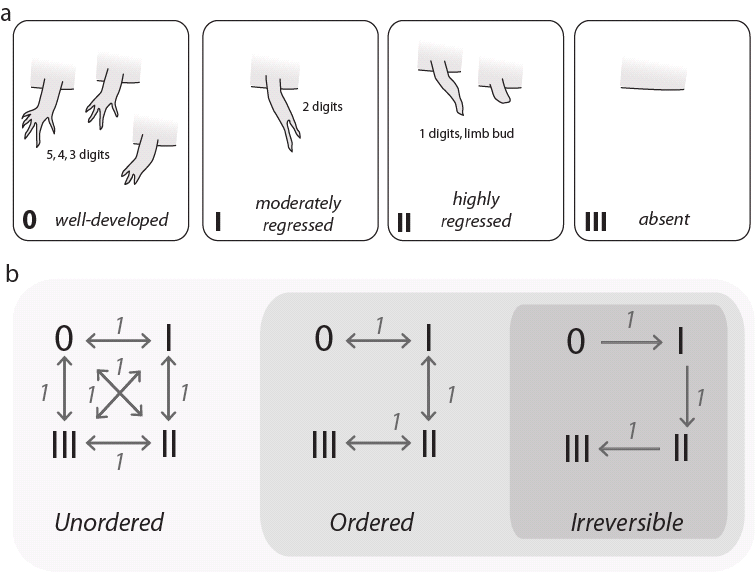


**S1B.** Encoding of the morphological data used for the analysis of character evolution. For each species, the limb morphology (number of digits when present, presence of a digitless limb bud, or absence of limb) is presented, followed in parenthesis by the corresponding “encoded state” used in the ancestral state reconstruction. Four different states have been encoded for both forelimbs and hindlimbs, according to their degree of regression: (0) *well-developed* (= presence of penta-, tetra- or tridactyl fore/hind-limbs), (I) *moderately regressed* (= presence of didactyl fore/hind-limbs), (II) *highly regressed* (= presence of monodactyl fore/hind-limbs or a digit-less limb-bud) and (III) *absent* (= absence of external fore/hind-limbs). Data from Andreone and Greer (2002), Glaw and Vences (2007) and the present study.

*For taxa presenting intraspecific polymorphism (variations of dactyly), only the most derived (regressed) state reached by these species has been retained and encoded (for *Pygomeles trivittatus*, the high intraspecific polymorphism observed corresponds to distinct populations regarded as two distinct subspecies by Brygoo (1987)).

| **Species** | **Fore limbs morphology**  **(encoding)** | **Hind limbs morphology**  **(encoding)** | |
| --- | --- | --- | --- |
| **Genus *“Amphiglossus”*** |  |  |  |
| *A. anosyensis* | pentadactyl (0) | pentadactyl (0) |  |
| *A. ardouini* | pentadactyl (0) | pentadactyl (0) |  |
| *A. astrolabi* | pentadactyl (0) | pentadactyl (0) |  |
| *A. crenni** | Two or three fingers (I) | Three or four fingers (0) |  |
| *A. frontoparietalis* | pentadactyl (0) | pentadactyl (0) |  |
| *A. macrocercus* | pentadactyl (0) | pentadactyl (0) |  |
| *A. mandokava* | pentadactyl (0) | pentadactyl (0) |  |
| *A. melanurus* | pentadactyl (0) | pentadactyl (0) |  |
| *A. meva* | pentadactyl (0) | pentadactyl (0) |  |
| *A. ornaticeps* | pentadactyl (0) | pentadactyl (0) |  |
| *A. punctatus* | pentadactyl (0) | pentadactyl (0) |  |
| *A. reticulatus* | pentadactyl (0) | pentadactyl (0) |  |
| *A. tanysoma* | pentadactyl (0) | pentadactyl (0) |  |
| *A. robustus* sp. | pentadactyl (0) | pentadactyl (0) |  |
| *A. phaeurus* sp. | pentadactyl (0) | pentadactyl (0) |  |
| *A. variegatus* sp. | pentadactyl (0) | pentadactyl (0) |  |
|  |  |  |  |
| **Genus *Madascincus*** |  |  |  |
| *M. arenicola* | pentadactyl (0) | pentadactyl (0) |  |
| *M. igneocaudatus s.s.* | pentadactyl (0) | pentadactyl (0) |  |
| *M.* sp. *igneocaudatus* | pentadactyl (0) | pentadactyl (0) |  |
| *M. polleni s.s.* | pentadactyl (0) | pentadactyl (0) |  |
| *M.* sp. *polleni* | pentadactyl (0) | pentadactyl (0) |  |
| *M. stumpffi* | pentadactyl (0) | pentadactyl (0) |  |
| *M. ankodabensis* | pentadactyl (0) | pentadactyl (0) |  |
| *M. melanopleura s.s.* | pentadactyl (0) | pentadactyl (0) |  |
| *M.* sp. *melanopleura* | pentadactyl (0) | pentadactyl (0) |  |
| *M. mouroundavae* | pentadactyl (0) | pentadactyl (0) |  |
| *M. baeus* sp.*** | Three or four fingers (0) | pentadactyl (0) |  |
|  |  |  |  |
| **Genus *Paracontias*** |  |  |  |
| *P. brocchii* | absent (III) | absent (III) |  |
| *P. hildebrandti* | absent (III) | absent (III) |  |
| *P. manify* | absent (III) | absent (III) |  |
| *P. minimus* | absent (III) | absent (III) |  |
| *P. rothschildi* | absent (III) | absent (III) |  |
| *P. fasika* | absent (III) | absent (III) |  |
| *P. kankana* | absent (III) | absent (III) |  |
| *P. vermisaurus* | absent (III) | absent (III) |  |
|  |  |  |  |
| **Genus *Pseudoacontias*** |  |  |  |
| *Ps. menamainty* | absent (III) | absent (III) |  |
|  |  |  |  |
| **Genus *Pygomeles*** |  |  |  |
| *Py. braconnieri* | absent (III) | Single toe (II) |  |
| *Py. petteri* | absent (III) | absent (III) |  |
| *Py. trivittatus** | Two to five fingers (I) | Two to five toes (I) |  |
|  |  |  |  |
| **Genus *Grandidierina*** |  |  |  |
| *G. fierinensis* (dark) | absent (III) | Two toes (I) |  |
| *G. fierinensis* (pale) | absent (III) | Two toes (I) |  |
| *G. lineata* | absent (III) | absent (III) |  |
| *G. petiti* | absent (III) | Single toe (II) |  |
| *G. rubrocaudata* | absent (III) | absent (III) |  |
|  |  |  |  |
| **Genus *Voeltzkowia*** |  |  |  |
| *V. mira* | absent (III) | absent (III) |  |
| *V. mobydick* | Fingerless limb bud (II) | absent (III) |  |
| *V. yamagishii* | Four fingers (0) | absent (III) |  |
|  |  |  |  |
| **Outgroup species** |  |  |  |
| *Eumeces* (outgroup) | pentadactyl (0) | pentadactyl (0) |  |
| *Tiliqua* (outgroup) | pentadactyl (0) | pentadactyl (0) |  |
|  |  |  |  |

**S1C.** Results of the ancestral state reconstructions using three different parsimony optimizations implemented in PAUP 4.0b10. (a, a’) unordered (Fitch parsimony), (b, b’) ordered (Wagner parsimony), and (c,c’) ordered and irreversible (Camin-Sokal parsimony). For each of these three approaches, analyses were performed using the ACCTRAN (a, b, c) and DELTRAN (a’, b’, c’) options. For each figure, character evolution is represented on the left side for the forelimbs (red gradient), and on the right side for the hindlimbs (blue gradient). Numbers in red and blue represent the number of character changes involved in each reconstruction, and the asterisks indicate reversals.


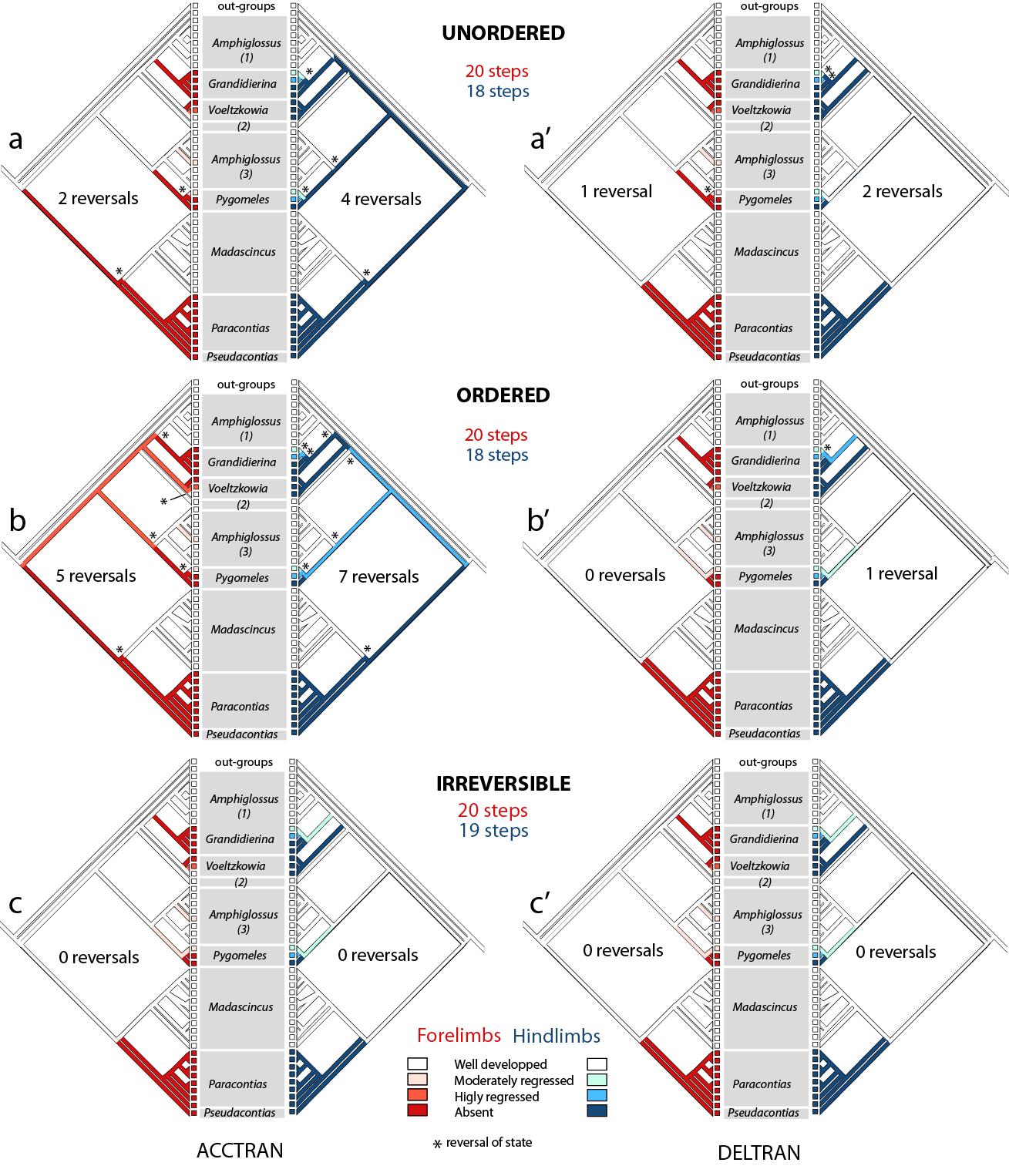


**References :**

Andreone F, Greer AE (2002) Malagasy scincid lizards: descriptions of nine new species, with notes on the morphology, reproduction and taxonomy of some previously described species (Reptilia, Squamata: Scincidae)*. Journal of Zoology (London), 258*, 139–181.

Brygoo E (1987) Systématique des lézards scincidés de la région malgache. XIX. Données nouvelles sur le genre *Androngo*. *Bulletin du Muséum National d'Histoire Naturelle, (Serie 4)*, *9*, 255–263.

Glaw F, Vences M (2007) *A field guide to the amphibians and reptiles of Madagascar. Third edition*. Cologne: Vences & Glaw Verlag.

Swofford DL (2002). PAUP*. Phylogenetic analysis using parsimony (and other methods). Version 4. Sinauer Associates, Sunderland, MA.
