# Supplementary material for: Distinct Patterns of Desynchronized Limb Regression in Malagasy Scincine Lizards (Squamata, Scincidae)
Source: PLoS One. 2015 Jun 4;10(6):e0126074. doi: 10.1371/journal.pone.0126074 (PMC4456255; doi:10.1371/journal.pone.0126074)

**S2 Appendix.** **Complementary phylogenetic analyses of Malagasy scincines.**

**S2A.** Bayesian analyses inferred from the mitochondrial DNA data set (12S, 16S and ND1) and the nuclear DNA data set (BDNF, RAG2, CMOS, PDC), with posterior probabilities.


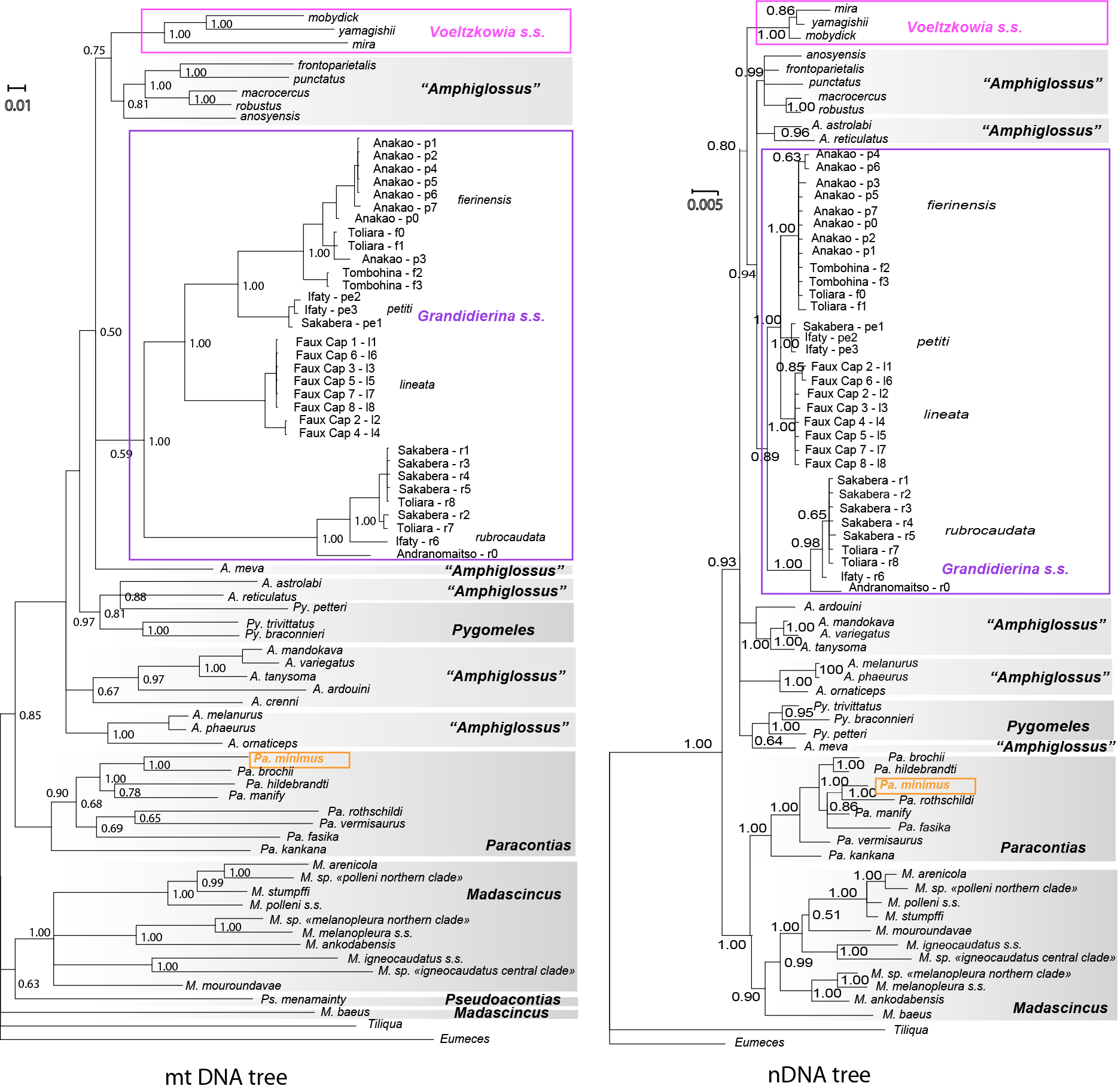


**S2B.** Maximum Parsimony analysis inferred from the complete DNA data set (12S, 16S, ND1, BDNF, RAG2, CMOS, PDC), with bootstrap support values indicated for each node >50% (CI=0.34, HI=0.66, RI=0.63, Tree length = 3822 steps, 2000 bootstrap replicates).


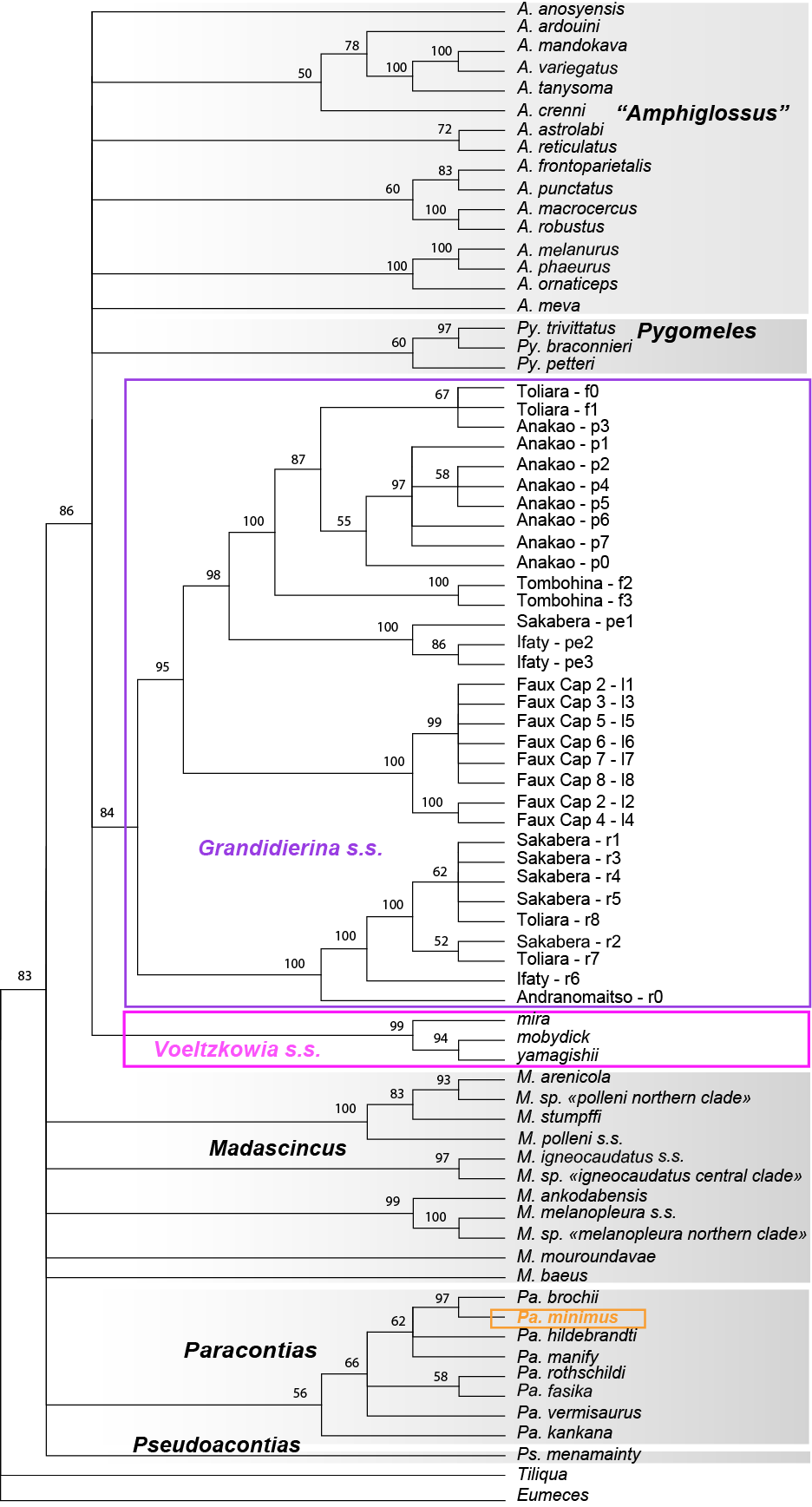

Supplement: S2 Appendix — (DOC) [file pone.0126074.s002.doc]
