# Supplementary material for: Distinct Patterns of Desynchronized Limb Regression in Malagasy Scincine Lizards (Squamata, Scincidae)
Source: PLoS One. 2015 Jun 4;10(6):e0126074. doi: 10.1371/journal.pone.0126074 (PMC4456255; doi:10.1371/journal.pone.0126074)
Supplement: S3 Appendix — (DOC) [file pone.0126074.s003.doc]

**S3 Appendix.** **Time trees of Malagasy scincines.**

Time trees of Malagasy scincines, using a Bayesian relaxed-clock phylogenetic approach, based on the whole dataset (mtDNA+nDNA) with a single prior for time calibration : (A) fixed mitochondrial substitution rate at 0.05/site/Mya and (B) with the divergence between *Madascincus* and *Amphiglossus* 47 Mya used for time calibration, as retrieved from a previous more comprehensive analysis of Malagasy vertebrates (Crottini *et al*. 2012). See caption of Fig. S4 for further explanation. Tree not used for further calculation due to the similarity of estimated divergence times with the two-priors approach (Fig. S4).

| 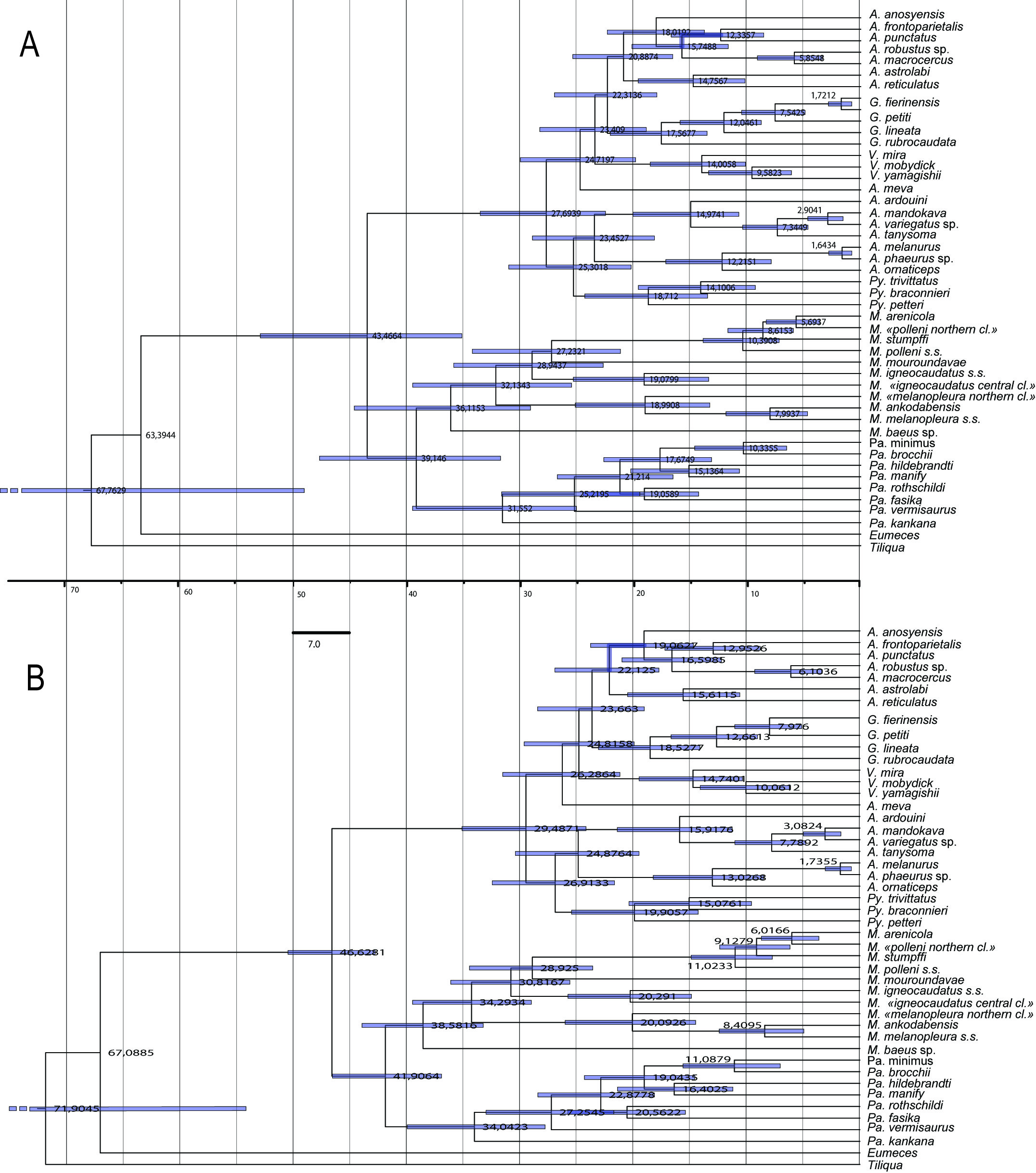 |
| --- |

**Reference :** Crottini, A., Dordel, J., Köhler, J., Glaw, F., Schmitz, A., & Vences, M. (2009). A multilocus phylogeny of Malagasy scincid lizards elucidates the relationships of the fossorial genera *Androngo* and *Cryptoscincus*. *Molecular Phylogenetics and Evolution, 53*, 345–350.
