# Supplementary material for: Distinct Patterns of Desynchronized Limb Regression in Malagasy Scincine Lizards (Squamata, Scincidae)
Source: PLoS One. 2015 Jun 4;10(6):e0126074. doi: 10.1371/journal.pone.0126074 (PMC4456255; doi:10.1371/journal.pone.0126074)
Supplement: S1 Table — (DOC) [file pone.0126074.s004.doc]

**S1 Table. List of voucher specimens, Genbank accession numbers and localities.**

Sequences newly generated for the present study are presented in bold (other sequences from Schmitz *et al*. 2005, Crottini *et al*. 2009, Miralles *et al*. 2011a,b,c, 2012, Miralles & Vences 2013). Missing data represented by dashes (—). Abbreviations: FG/MV, FGZC and ZCMV refer to Frank Glaw and Miguel Vences field numbers; MVTIS refers to the tissue collection of M. Vences; DRV refers to David R. Vieites field numbers; AGZC refers to field numbers of Angelica Crottini; MNCN, Museo Nacional de Ciencias Naturales, Madrid; MRSN, Museo Regionale di Scienze Naturali, Torino; UADBA, Université d’Antananarivo, Département de Biologie Animale (partly not yet catalogued); ZMA, Zoölogisch Museum Amsterdam; ZSM, Zoologische Staatssammlung München.

| **Species** | **Locality** | **Voucher** |  | **12S** | **16S** | **ND1** | **BDNF** | **CMOS** | **RAG2** | **PDC** |
| --- | --- | --- | --- | --- | --- | --- | --- | --- | --- | --- |
| ***« Amphiglossus »*** |  |  |  |  |  |  |  |  |  |  |
| *anosyensis* | Ambatolahy | ZCMV 591  (ZMA 20342) |  | FJ667609 | FJ667621 | FJ744569 | FJ667634 | FJ667663 | FJ667721 | FJ667692 |
| *ardouini* | Montagne des Français | —  (FGZC 1721) |  | — | **KM057145** | **KM057252** | **KM057180** | **KM057216** | **KM057323** | **KM057288** |
| *astrolabi* | Ranomafana | ZSM 201/2003  (FG/MV 2002-312) |  | AY315474 | AY315523 | FJ744570 | FJ667635 | FJ667664 | FJ667722 | FJ667693 |
| *crenni* | Andasibe | ZSM 288/2002 |  | AY315504 | AY315553 | AY315599 | — | — | — | — |
| *frontoparietalis* | Ambohitsara | ZMA 20341  (ZCMV 153) |  | FJ667610 | FJ667622 | FJ744571 | FJ667636 | FJ667665 | FJ667723 | FJ667694 |
| *macrocercus* | Ankaratra | ZSM 016/2003  (FG/MV 2002-2142) |  | AY315484 | AY315533 | FJ744572 | FJ667637 | FJ667666 | FJ667724 | FJ667695 |
| *mandokava* | Montagne d’Ambre | ZSM 2167/2007  (FGZC 1240) |  | FJ667611 | FJ667623 | FJ744573 | FJ667638 | FJ667667 | FJ667725 | FJ667696 |
| *melanurus* | Maroantsetra | —  (MVTIS 2002-A6) |  | AY315502 | AY315551 | FJ744574 | FJ667639 | FJ667668 | FJ667726 | FJ667697 |
| *meva* | Makira | MNCN 44649  (DRV 5885) |  | JF424673 | JF424678 | JF424703 | JF424687 | JF424692 | JF424717 | JF424711 |
| *ornaticeps* | Ifaty Mangily Reserve | ZSM 1603/2010(ZCMV 13010) |  | — | **KM057146** | **KM057253** | **KM057181** | **KM057217** | **KM057324** | **KM057289** |
| *punctatus* | Ambatolahy | ZMA 20230  (ZCMV 519) |  | AY315489 | FJ667624 | FJ744575 | FJ667640 | FJ667669 | FJ667727 | FJ667698 |
| *reticulatus* | Berara | —  (MVTIS 2000-E44) |  | AY315490 | AY315539 | FJ744576 | FJ667641 | FJ667670 | FJ667728 | FJ667699 |
| *tanysoma* | Berara | —  (MVTIS 2000-D58) |  | AY315498 | AY315547 | FJ744577 | FJ667642 | FJ667671 | FJ667729 | FJ667700 |
| sp. *‘‘robustus”* | Andasibe | ZMA 20228  (ZCMV 373) |  | FJ667612 | FJ667625 | FJ744562 | FJ667643 | FJ667672 | FJ667730 | FJ667701 |
| sp. *‘‘phaeurus”* | Andasibe | UADBA uncat.  (ZCMV 3062) |  | FJ667613 | FJ667626 | FJ744563 | FJ667644 | FJ667673 | FJ667731 | FJ667702 |
| sp. *‘‘variegatus”* | Montagne des Francais | ZSM 246/2004  (FGZC 482) |  | FJ667614 | FJ667627 | FJ744564 | FJ667645 | FJ667674 | FJ667732 | FJ667703 |
| ***« Madascincus »*** |  |  |  |  |  |  |  |  |  |  |
| *arenicola* | Baie des Sakalava | ZSM 1568/2008  (FGZC 1767) |  | — | JQ007911 | JQ008374 | JQ008065 | JQ008221 | JQ008393 | JQ008541 |
| *igneocaudatus (Southern clade)* | Faux Cap | ZSM 1601/2010 (ZCMV 12897) |  | — | JQ007920 | JQ008383 | JQ008074 | JQ008229 | JQ008402 | JQ008550 |
| *igneocaudatus (Central clade)* | Ibity | —  (MVTIS 2001-D14) |  | AY315476 | FJ667629 | FJ744567 | FJ667648 | FJ667677 | FJ667735 | FJ667706 |
| *melanopleura N* | An'Ala forest camp | ZSM 207/2006  (ZCMV 2481) |  | — | JQ007926 | JQ008689 | JQ008080 | JQ008235 | JQ008408 | JQ008556 |
| *melanopleura C* | Andasibe | ZSM 14/2005  (ZCMV 2258) |  | — | JQ007939 | JQ008701 | JQ008093 | JQ008248 | JQ008420 | JQ008567 |
| *melanopleura S* | Ambatolahy river | ZSM 354/2006  (ZCMV 3061) |  | — | JQ007923 | JQ008686 | JQ008077 | JQ008232 | JQ008405 | JQ008553 |
| *mouroundavae* | Antsahamanara | —  (MVTIS 2001-F17) |  | AY315487 | AY315536 | FJ744578 | FJ667650 | FJ667679 | FJ667737 | FJ667708 |
| *polleni (Southern clade)* | Ankarafantsika (Ampijoroa) | —  (MVTIS 2001-B55) |  | AY315479 | AY315528 | FJ744568 | FJ667649 | FJ667678 | FJ667736 | FJ667707 |
| *polleni (Northern clade)* | Montagne des Francais | ZSM 245/2004  (FGZC 0480) |  | — | JQ008028 | JQ008787 | JQ008182 | JQ008338 | JQ008506 | JQ008656 |
| *stumpffi* | Forêt d'Ambre | ZSM 1558/2008  (FGZC 3124) |  | — | JQ008034 | JQ008793 | JQ008189 | JQ008345 | JQ008512 | JQ008663 |
| sp. *‘‘baeus”* | Andasibe | UADBA uncat  (ZCMV 2283) |  | FJ667617 | AY315542 | FJ744580 | FJ667652 | FJ667681 | FJ667739 | FJ667710 |
| ***Paracontias*** |  |  |  |  |  |  |  |  |  |  |
| *brocchii* | Montagne d’Ambre | ZSM 244/2004  (FGZC 476) |  | AY315507 | AY391155 | FJ744583 | FJ667655 | FJ667684 | FJ667742 | FJ667713 |
| *fasika* | Baie de Sakalava | ZSM 2256/2007  (FGZC 1347) |  | FJ667619 | FJ667632 | FJ744589 | FJ667661 | FJ667690 | FJ667748 | FJ667719 |
| *hildebrandti* | Montagne des Francais | ZSM 1578/2008  (FGZC 1946) |  | FJ667620 | FJ667633 | FJ744590 | FJ667662 | FJ667691 | FJ667749 | FJ667720 |
| *kankana* | Mahasoa forest | ZSM 1810/2008  (DRV 5711) |  | AY315509 | FJ667631 | FJ744582 | FJ667654 | FJ667683 | FJ667741 | FJ667712 |
| *manify* | Antsahamanara | MRSN R 1887  (MVTIS 2001-F58) |  | AY315510 | AY315559 | FJ744584 | FJ667656 | FJ667685 | FJ667743 | FJ667714 |
| *.minimus* | Baie de Sakalava | ZSM 2251/2007  (FGZC 1027 ) |  | FJ667616 | FJ667628 | FJ744566 | FJ667647 | FJ667676| | FJ667734 | FJ667705 |
| *rothschildi* | Baie de Sakalava | ZSM 2246/2007  (FGZC 1020) |  | FJ667618 | FJ667630 | FJ744581 | FJ667653 | FJ667682 | FJ667740 | FJ667711 |
| *vermisaurus* | Makira | ZSM 0597/2008  (ZCMV 11211) |  | — | HQ891855 | HQ891854 | HQ891856 | — | HQ891858 | HQ891857 |
| ***Pseudoacontias*** |  |  |  |  |  |  |  |  |  |  |
| *menamainty* | Berara Forest | MRSN R1826 |  | AY315511 | AY315560 | AY315606 | — | — | — | — |
| ***Pygomeles*** |  |  |  |  |  |  |  |  |  |  |
| *braconnieri* | Ifaty | —  (FG/MV 2002-2048) |  | AY315514 | AF215235 | FJ744585 | FJ667657 | FJ667686 | FJ667744 | FJ667715 |
| *petteri* | Ankarafantsika National Park | ZSM 3206/2012  (ZCMV 14104) |  | **KM057077** | **KM057111** | **KM057218** | **KM057147** | **KM057182** | **KM057290** | **KM057254** |
| *trivittatus* | Tolagnaro | ZSM 389/2005  (FGZC 2306) |  | FJ667615 | AY151444 | FJ744565 | FJ667646 | FJ667675 | FJ667733 | FJ667704 |
| ***Grandidierina*** |  |  |  |  |  |  |  |  |  |  |
| *fierinensis* | Arboretum of Toliara | UADBA uncat. (FG/MV 2000-569) | f0 | AY315516 | AY315563 | FJ744586 | FJ667658 | FJ667687 | FJ667745 | FJ667716 |
|  | Near Toliara | ZSM 386/2005  (FGZC 2685) | f1 | **KM057078** | **KM057112** | **KM057219** | **KM057148** | **KM057183** | **KM057291** | **KM057255** |
|  | Tombohina | ZSM 1618/2010 (ZCMV 12887) | f2 | **KM057079** | **KM057113** | **KM057220** | **KM057149** | **KM057184** | **KM057292** | **KM057256** |
|  | Tombohina | ZSM 1619/2010 (ZCMV 12884) | f3 | **KM057080** | **KM057114** | **KM057221** | **KM057150** | **KM057185** | **KM057293** | **KM057257** |
|  | Anakao | —  (ZCMV 5539 ) | p1 | **KM057081** | **KM057115** | **KM057222** | **KM057151** | **KM057186** | **KM057294** | **KM057258** |
|  | Anakao | UADBA uncat. (ZCMV12880) | p2 | **KM057082** | **KM057116** | **KM057223** | **KM057152** | **KM057187** | **KM057295** | **KM057259** |
|  | Anakao | UADBA uncat. (ZCMV12881) | p3 | **KM057083** | **KM057117** | **KM057224** | **KM057153** | **KM057188** | **KM057296** | **KM057260** |
|  | Anakao | ZSM 1633/2010 (ZCMV12882) | p4 | **KM057084** | **KM057118** | **KM057225** | **KM057154** | **KM057189** | **KM057297** | **KM057261** |
|  | Anakao | ZSM 1634/2010 (ZCMV12883) | p5 | **KM057085** | **KM057119** | **KM057226** | **KM057155** | **KM057190** | **KM057298** | **KM057262** |
|  | Anakao | ZSM 1635/2010 (ZCMV12885) | p6 | **KM057086** | **KM057120** | **KM057227** | **KM057156** | **KM057191** | **KM057299** | **KM057263** |
|  | Anakao | ZSM 1636/2010 (ZCMV12886) | p7 | **KM057087** | **KM057121** | **KM057228** | **KM057157** | **KM057192** | **KM057300** | **KM057264** |
|  | Anakao | UADBA uncat.  (FG/MV 2002-1536) | p8 | AY315589 | AY315565 | FJ744588 | FJ667660 | FJ667689 | FJ667747 | FJ667718 |
| *lineata* | Faux Cap | ZSM 1621/2010 (ZCMV 12894) | l1 | **KM057088** | **KM057122** | **KM057229** | **KM057158** | **KM057193** | **KM057301** | **KM057265** |
|  | Faux Cap | ZSM 1622/2010  (ZCMV 12893) | l2 | **KM057089** | **KM057123** | **KM057230** | **KM057159** | **KM057194** | **KM057302** | **KM057266** |
|  | Faux Cap | ZSM 1623/2010  (ZCMV 12891) | l3 | **KM057090** | **KM057124** | **KM057231** | **—** | **KM057195** | **KM057303** | **KM057267** |
|  | Faux Cap | ZSM 868/2010  (ZCMV 12846) | l4 | **KM057091** | **KM057125** | **KM057232** | **KM057160** | **KM057196** | **KM057304** | **KM057268** |
|  | Faux Cap | ZSM 1624/2010  (ZCMV 12845) | l5 | **KM057092** | **KM057126** | **KM057233** | **KM057161** | **KM057197** | **KM057305** | **KM057269** |
|  | Faux Cap | ZSM 1625/2010  (ZCMV 12850) | l6 | **KM057093** | **KM057127** | **KM057234** | **KM057162** | **KM057198** | **KM057306** | **KM057270** |
|  | Faux Cap | ZSM 1626/2010 (ZCMV 12847) | l7 | **KM057094** | **KM057128** | **KM057235** | **KM057163** | **KM057199** | **KM057307** | **KM057271** |
|  | Faux Cap | —  (ZCMV 12848) | l8 | **KM057095** | **KM057129** | **KM057236** | **KM057164** | **KM057200** | **KM057308** | **KM057272** |
| *petiti* | Sakabera, near Ifaty | ZSM 1620/2010  (ZCMV 12824) | pe1 | **KM057096** | **KM057130** | **KM057237** | **KM057165** | **KM057201** | **KM057309** | **KM057273** |
|  | Ifaty Mangily Reserve | ZSM 1617/2010 (ZCMV 13009) | pe2 | **KM057097** | **KM057131** | **KM057238** | **KM057166** | **KM057202** | **KM057310** | **KM057274** |
|  | Ifaty | ZSM 228/2003  (FG/MV 2002- 2051) | pe3 | **KM057098** | **KM057132** | **KM057239** | **KM057167** | **KM057203** | **KM057311** | **KM057275** |
| *rubrocaudata* | Sakabera, near Ifaty | ZSM 1628/2010  (ZCMV 12832) | r1 | **KM057099** | **KM057133** | **KM057240** | **KM057168** | **KM057204** | **KM057312** | **KM057276** |
|  | Sakabera, near Ifaty | ZSM 1629/2010  (ZCMV 12833) | r2 | **KM057100** | **KM057134** | **KM057241** | **KM057169** | **KM057205** | **KM057313** | **KM057277** |
|  | Sakabera, near Ifaty | ZSM 1630/2010  (ZCMV 12830) | r3 | **KM057101** | **KM057135** | **KM057242** | **KM057170** | **KM057206** | **KM057314** | **KM057278** |
|  | Sakabera, near Ifaty | ZSM 1631/2010  (ZCMV 12829) | r4 | **KM057102** | **KM057136** | **KM057243** | **KM057171** | **KM057207** | **KM057315** | **KM057279** |
|  | Sakabera, near Ifaty | ZSM 1632/2010 (ZCMV 12831) | r5 | **KM057103** | **KM057137** | **KM057244** | **KM057172** | **KM057208** | **KM057316** | **KM057280** |
|  | Ifaty | ZSM 232/2003  (FG/MV 2002- 2050) | r6 | **KM057104** | **KM057138** | **KM057245** | **KM057173** | **KM057209** | **KM057317** | **KM057281** |
|  | Toliara | ZSM 384/2005  (FGZC 2683) | r7 | **KM057105** | **KM057139** | **KM057246** | **KM057174** | **KM057210** | **KM057318** | **KM057282** |
|  | Toliara | ZSM 385/2005  (FGZC 2684) | r8 | **KM057106** | **KM057140** | **KM057247** | **KM057175** | **KM057211** | **KM057319** | **KM057283** |
|  | Andranomaitso, Sakaraha | MRSN R3726  (AGZC 2565) | r0 | **KM057107** | **KM057141** | **KM057248** | **KM057176** | **KM057212** | **—** | **KM057284** |
| ***Voeltzkowia*** |  |  |  |  |  |  |  |  |  |  |
| *mira* | Antsanitia | —  (ZCMV 14110) |  | **KM057108** | **KM057142** | **KM057249** | **KM057177** | **KM057213** | **KM057320** | **KM057285** |
| *mobydick* |  | ZSM 3207/2012  (ZCMV 13587) |  | **KM057109** | **KM057143** | **KM057250** | **KM057178** | **KM057214** | **KM057321** | **KM057286** |
| *yamagishii* | Ankarafantsika National Park | —  (ZCMV 14103) |  | **KM057110** | **KM057144** | **KM057251** | **KM057179** | **KM057215** | **KM057322** | **KM057287** |
| **Out groups** |  |  |  |  |  |  |  |  |  |  |
| *’’Eumeces” sensu lato spp.* |  | —  — |  | EU278021 | EU278085 | AY315600 | EF646320 | EF646320 | DQ119628 | — |
| *Tiliqua sp.* |  | —  — |  | AB057376 | AY217965 | — | — | — | EF534983 | EF534856 |

**References :**

### Crottini A, Madsen O, Poux C, Strau**ß** A, Vieites DR, Vences M (2012) Vertebrate time-tree elucidates the biogeographic pattern of a major biotic change around the K–T boundary in Madagascar. *Proceedings of the National Academy of Sciences* *USA,* *109*, **5358–5363.**

**Miralles A**, Köhler J, Vieites DR, Glaw F, Vences M (2011a) Developing hypotheses on rostral shield evolution in head-first digging squamates from a molecular phylogeny and new species. *Organisms Diversity and Evolution*, *11*, 135–150.

**Miralles A**, Köhler J, Glaw F, Vences M (2011b) A molecular phylogeny of the *Madascincus polleni* species complex, with description of a new species of scincid lizard from the coastal dune area of northern Madagascar. *Zootaxa*, *2876*, 1–16.

Miralles A, Raselimanana AP, Rakotomalala D, Vences M, Vieites DR (2011c) A new large and colorful skink of the genus *Amphiglossus* from Madagascar revealed by morphology and multilocus molecular study. *Zootaxa*, 2918, 47–67.

**Miralles A**, Anjeriniana M, Hipsley CA, Mueller J, Glaw F, Vences M (2012) Variations on a bodyplan: description of a new Malagasy “mermaid skink” with flipper-like forelimbs only (Scincidae: *Sirenoscincus*). *Zoosystema*, *34,* 701–710.

Miralles A, Vences M (2013) New metrics for comparison of taxonomies reveal striking discrepancies among species delimitation methods in *Madascincus* lizards. *PloS ONE*, *8*, e68242. doi: 10.1371/journal.pone.0068242

Schmitz A, Brandley MC, Mausfeld P, Vences M, Glaw F, Nussbaum RA, Reeder TW (2005) Opening the black box: phylogenetics and morphological evolution of the Malagasy fossorial lizards of the subfamily ‘‘Scincinae’’. *Molecular Phylogenetics and Evolution*, *34*, 118–133.
