# Supplementary material for: Distinct Patterns of Desynchronized Limb Regression in Malagasy Scincine Lizards (Squamata, Scincidae)
Source: PLoS One. 2015 Jun 4;10(6):e0126074. doi: 10.1371/journal.pone.0126074 (PMC4456255; doi:10.1371/journal.pone.0126074)
Supplement: S1 Text — (DOC) [file pone.0126074.s005.doc]

**S1 Text. Osteological descriptions.**

***Amphiglossus ornaticeps*** (ZSM 1603/2010=ZCMV 13010)*.* ─ **External features.** A relatively elongated small-sized skink with four relatively well developed pentadactyl limbs, each fingers and toes being clawed. **Pectoral girdle.** Complete and well developed, the pectoral girdle is dorso-ventrally flattened and roughly hexagonal, approximately as long as wide. Clavicles are strongly curved ventrally (S-shaped) with a thin and regular rounded transversal section, with apparently both proximal and distal extremity rounded, and a small and hardly visible process at their mid-length, posteriorly directed. The interclavicle is cruciform, with an apparently round-ended anterior process approximately as long as the lateral processes which have narrow and pointed distal ends, and a round-ended posterior process about one and a half times longer. Suprascapulae are triangular, more ossified medially than laterally. Scapula, coracoid and precoracoid are not well distinguishable from each other, forming a continuous scapulocoracoid bone. The pericoracoid is extremely regressed, fragmented into several poorly ossified residues: two stripes separating the sternum from the coracoid, and two pairs of small rodlike structures, posteriorly barely contacting with the cranial extremities of the precoracoid and the coracoid, respectively, and anteriorly converging toward the anterior part of the interclavicle. The coracoid foramen is small, oval, and almost open into the anterior coracoid fenestra. Both the anterior (= primary) and posterior (= secondary) coracoid fenestra are not completely closed, the pericoracoid being too reduced to delimitate their anterior margin. The kite-shaped sternum is relatively well ossified, as long as wide, more ossified laterally than medially, without median fontanel, and laterally connected to three pairs of sternal ribs. The xiphisternum is “arrow-shaped”, with four elongated rod-like processes: an antero-median process connecting the posteriormost extremity of the sternum, a pair of posterolaterally directed processes connecting the first pair of xiphisternal ribs, and a postero-median process oriented backward and connecting the second pair of xiphisternal ribs. **Forelimbs.** Forelimbs are pentadactyl and well-developed. The humerus is elongated, articulating with the scapulocoracoid through a well-developed glenoid fossa, and with enlarged proximal and distal ends twisted in relation to one another at an angle of approximately 90°. Ulna and radius are relatively reduced in comparison to the humerus, as they approximately represent only two thirds of its length. The manus shows no reductions in carpus and metacarpus, and the phalangeal formula is 2-3-3-4-3. **Pelvic girdle.** Well developed, the pelvic girdle consists of two partly fused trifurcated hemipelves, each composed of three elongated projections which are the pubis, ischium and ilium, sharing laterally a sutural union centred on the acetabulum. Pubes are rodlike structures with a hook-shaped ventral process oriented backward, craniomedially converging and suturing into a small glomerular epipubis. Ischia are flattened structures with a small caudally oriented process, and are medioventrally converging and suturing. Ilia are elongated parallel rodlike structures oriented dorsocaudally. **Hindlimbs.** Hindlimbs are pentadactyl and well developed. The femur is elongated, articulating with the pelvis through a relatively well developed actetabular fossa, and with enlarged proximal and distal ends. Tibia and fibula are slightly reduced in comparison to the femur, as they approximately represent only two thirds of its length. The pes shows no signs of reduction and the phalangeal formula is 2-3-4-5-4.

***Paracontias minimus*** (ZSM 2253/2007). **─ External features.** An elongated and slender, small-sized blind skink without any visible external limbs. **Pectoral girdle.** Highly regressed, the pectoral girdle is dorso-ventrally flattened, two times wider than long, and chevron-shaped. Clavicles, which are apparently locally fused at mid-length to the scapulocoracoid, are highly regressed, with a flattened and poorly ossified curved proximal half, and a straight rode-like distal half running along the scapulocoracoid bone and the coracoid process of the scapula. The interclavicle is small, flat and triangular in shape with a very short posterior process. Suprascapulae are roughly rectangular, more ossified medially than laterally and with a narrower coracoid process oriented ventrally, in continuity with the longitudinal axis of the scapulocoracoid bone axis. Scapula, coracoid and precoracoid cannot be distinguished from each other, forming a very regressed and compact scapulocoracoid bone without foramina nor fenestra, with an enlarged and ventro-dorsally flattened proximal extremity contacting the sternum and a narrower sub-cylindrical distal extremity contacting the scapula. The pericoracoid is apparently completely regressed. The rhombhoedric sternum is well ossified, without a median fontanel, and in contact with two pairs of sternal ribs, one pair connected laterally and one pair connected to the posterior tip. Absence of xiphisternum. **Forelimbs.** Completely regressed, no vestigial bones. **Pelvic girdle.** Highly reduced, the pelvic girdle is composed of two separate elongated and curved hemipelves, without acetabular depression. Pubis and ischium are ventro-laterally flattened and distally diverging, forming a V-shape bifurcated ventro-cranial end of each hemipelvis, whereas the ilium forms an elongated cigar-shaped dorso-caudal projection. **Hindlimbs.** Completely regressed, no vestigial bones.

***Grandidierina fierinensis*** (ZSM 1636/2010) **─ External features.** An elongated and slender, small-sized blind skink with two poorly developed hindlimbs, with only two clawed toes; no visible external forelimbs. **Pectoral girdle.** Highly regressed, the pectoral girdle is dorso-ventrally flattened, two time wider than long, roughly hexagonal, and with a very compacted overall aspect (all its bones are adpressed against the others, assembled together on the same plane. Clavicles are relatively straight, flattened dorso-ventrally, with an enlarged and flattened triangular proximal extremity, poorly ossified at their centre, and a narrow and elongated distal extremity running along the scapulocoracoid bone and the coracoid process of the scapula. The interclavicle is highly regressed, flat and rounded. Suprascapulae are roughly fan-shaped and flattened, with an enlarged body extending dorsally and curved backward, and with a narrower coracoid process oriented ventrally, in continuity with the longitudinal axis of the scapulocoracoid bone axis. Scapula, coracoid and precoracoid cannot be distinguished from each other, forming a very regressed and compact rode-like scapulocoracoid bone without foramina nor fenestra, sub-cylindrical in section, and with slightly enlarged ventro-dorsally flattened extremities contacting the suprascapula dorsally and the sternum ventrally. The pericoracoid is apparently completely regressed. The roughly rectangular sternum is well ossified, two times wider than long, without median fontanel, and laterally connected to a single pair of sternal ribs. Absence of xiphisternum. **Forelimbs.** Completely regressed, no vestigial bones. **Pelvic girdle.** Moderately regressed, the pelvic girdle consists in two trifurcated hemipelves in contact - but not fused - with each other, each composed by three elongated projections which are the pubis, ischium and ilium, sharing laterally a sutural union centred on the acetabulum. Pubes are rodlike structures with a small hook-shaped ventral process oriented backward, craniomedially converging and barely contacting each other, and their extremities are preceded by two small glomerular residues of the epipubis. Ischia are flattened structures with a small laterocaudally oriented process, and are medioventrally converging but not contacting each other. Ilia are parallel and elongated rodlike structures oriented dorsocaudally. **Hindlimbs.** Hindlimbs are significantly regressed, with two digits (III and IV) only. The femur is elongated, articulating with the pelvis through a moderately developed acetabular fossa, and with enlarged proximal and distal ends. Tibia and fibula are relatively reduced in comparison to the humerus, as they approximately represent only half of its length. The reduced pes consists of astragalus and calcaneum, 2 distal tarsals, 2 metatarsals, and 2 digits with 2 and 3 phalangeal elements, respectively. Based on its distinctly hooked shape, one of the metatarsals can be tentatively identified as metatarsal 5, i.e. the 5th digit is present; the other digit is therefore most likely digit 4. From this follows that the 2 distal tarsals are probably distals tarsal 4 and 3.

***Grandidierina lineata*** (ZSM 1624/2010) **─ External features**. An elongated and slender, small-sized blind skink without any visible external limbs. **Pectoral girdle.** Relatively regressed, the pectoral girdle is dorso-ventrally flattened, almost two times wider than long, roughly rhomboidal and with a very compacted overall aspect (all its bones are adpressed against the others, assembled together in the same plane. Clavicles are relatively straight, flattened dorso-ventrally, with an enlarged and flattened rounded proximal extremity curved cranially, and a narrow and elongated distal extremity, running along the scapulocoracoid bone and the coracoid process of the scapula. The interclavicle is lozenge-shaped, slightly longer than wide, the anterior, lateral and to a less extent the posterior processes being highly regressed. Suprascapulae are roughly rectangular, more ossified medially than laterally and with a narrower coracoid process oriented ventrally, in continuity with the longitudinal axis of the scapulocoracoid bone axis. Scapula, coracoid and precoracoid cannot be distinguished from each other, forming a very regressed and compact spatulated scapulocoracoid bone without foramina nor fenestra, sub-cylindrical in section, and with an enlarged ventro-dorsally flattened proximal extremity contacting the sternum and a narrow distal extremity contacting the suprascapula. The pericoracoid is apparently completely regressed. The rhombohedric sternum is very poorly ossified medially (although the sternal fontanel can hardly be delimited), as long as wide, and postero-laterally connected to two pairs of sternal ribs. Absence of xiphisternum. **Forelimbs.** Completely regressed, no vestigial bones. **Pelvic girdle.** Highly reduced, the pelvic girdle is composed of two separate, curved and rodlike hemipelves, without acetabular depression. Pubis and ischium are completely fused, hardly distinguishable from each other, forming the anteroventral projection of each hemipelvis, laterally compressed and curved, with a long and pointed ventro-cranial end, whereas the ilium forms the elongated and pointed dorso-caudal projection. Hindlimbs. Completely regressed, no vestigial bones.

***Grandidierina petiti*** (ZSM 1620/2010) **─ External features.** An elongated and slender, small-sized blind skink with two minute stick-like hindlimbs, extremely regressed, and without any differentiated toes nor claws; no visible external forelimbs. **Pectoral girdle.** Relatively regressed, the pectoral girdle is dorso-ventrally flattened, slightly wider than long, and roughly lozenge-shaped. Clavicles are flattened dorso-ventrally, with a very enlarged and flattened triangular proximal extremity, and a narrow and elongated distal extremity running along the scapulocoracoid bone and the coracoid process of the scapula. The interclavicle is highly regressed, anteroposteriorly elongated with pointed extremities, the lateral processes are completely regressed. Suprascapulae are elongated and flattened, roughly trapezoidal, more ossified medially than laterally, and with a narrower coracoid process oriented ventrally, in continuity with the longitudinal axis of the scapulocoracoid bone axis. Scapula, coracoid and precoracoid cannot be distinguished from each other, forming a very regressed and compact spatulated scapulocoracoid bone without foramens nor fenestra, sub-cylindrical in section, and with an enlarged ventro-dorsally flattened proximal extremity contacting the sternum and a narrow distal extremity contacting the suprascapula. The pericoracoid is apparently completely regressed. Roughly rectangular, the sternum is poorly ossified, two times wider than long, very poorly ossified medially (although the sternal fontanel can hardly be delimited), and laterally connected to a single pair of sternal ribs. Absence of xiphisternum. **Forelimbs.** Completely regressed, no vestigial bones. **Pelvic girdle.** Moderately regressed, the pelvic girdle consists in two trifurcated hemipelves barely in contact - but not fused - with each other, each composed by three elongated projections which are the pubis, ischium and ilium, sharing laterally a sutural union centred on the acetabulum. Pubes are rodlike structures with a small ventral process, craniomedially converging and barely contacting each other, and their extremities are preceded by an extremely reduced median residue of the epipubis. Relatively reduced, ischia are flattened structures with a small caudally oriented process, and are medioventrally converging but not contacting each other. Ilia are parallel and elongated rodlike structures oriented dorsocaudally. **Hindlimbs.** Hindlimbs are significantly regressed, with a single digit (likely finger IV) only. The femur is elongated, articulating with the pelvis through a moderately developed acetabular fossa, and with enlarged proximal and distal ends. Tibia and fibula are relatively reduced in comparison to the humerus, as they represent a bit less than half of its length. The pes is strongly reduced, consisting of a small astragalus and calcaneum, a lateral tarsal element the identity of which cannot be identified, one metatarsal and one phalangeal element. The stout appearance of the metatarsal might suggest it is metatarsal 5, but this interpretation must remain speculative.

***Grandidierina rubrocaudata*** (ZSM 1632/2010) **─ External features.** An elongated and slender, small-sized blind skink without any visible external limbs. **Pectoral girdle.** Highly regressed, the pectoral girdle is dorso-ventrally flattened and roughly triangular, almost two times wider than long, and with a relatively compacted overall aspect. Clavicles are flattened dorso-ventrally, with an elongated 90° curved proximal extremity posteriorly directed, a narrow and pointed distal extremity, and a reduced process at their mid-length posteriorly directed. Absence of interclavicle. Suprascapulae are roughly fan-shaped and flattened, with an enlarged body extending dorsally and curved backward, and with a narrower coracoid process oriented ventrally, in continuity with the longitudinal axis the scapulocoracoid bone. Scapula, coracoid and precoracoid cannot be distinguished from each other, forming a very regressed and compact spatulated scapulocoracoid bone without foramina nor fenestra, dorso-ventrally flattened, and with an enlarged proximal extremity contacting the sternum and a narrower distal extremity contacting the suprascapula. The pericoracoid is almost completely regressed, with very weak residues separating the sternum from the coracoid. The pentagonal sternum is well ossified, slightly longer than wide, without median sternal fontanel, and laterally connected to a single pair of sternal ribs. Absence of xiphisternum. **Forelimbs.** Completely regressed, no vestigial bones. **Pelvic girdle.** Highly reduced, the pelvic girdle is composed of two separate, elongated and straight hemipelves, with a poorly depressed acetabulur depression. Pubis and ischium are laterally flattened, and form the anteroventral projection of each hemipelvis. Their proximal and distal parts are fused, but not their respective intermediate segments, which therefore delimitate an elliptic ischio-pubial fenestra. The ilium forms an elongated and laterally flattened dorsal projection. **Hindlimbs.** Hindlimbs bones are almost completely regressed as it only subsists a pair of extremely reduced pear-shaped femur articulating with the pelvis through a relatively well developed acetabular fossa.

***Voeltzkowia mira*** (ZSM 867/0) **─ External features.** An elongated and slender, small-sized blind skink without any visible external limbs, but with the presence on the flanks of two small areas where scales are reduced, indicating the former position of forelimb insertions. **Pectoral girdle.** Complete and relatively well developed, the pectoral girdle is dorso-ventrally flattened and roughly rhomboidal, approximately as long as wide. Clavicles are strongly curved (S-shaped) with a thin and regular rounded transversal section at mid-length, a flattened and rounded proximal extremity, a narrow and pointed distal extremity going along the coracoid process of the scapula, and a reduced process at their mid-length posteriorly directed. The interclavicle is cruciform, with four processes approximately subequal in length. Suprascapulae are elongated and flattened, with an enlarged marginated distal part and with a narrower coracoid process oriented ventrally, in continuity with the longitudinal axis of the scapulocoracoid bone axis. Scapula, coracoid and precoracoid are not distinct from each other, forming a continuous scapulocoracoid bone. The pericoracoid is extremely regressed, fragmented into several poorly ossified residues: two stripes separating the sternum from the coracoid, and one pair of small elongated structures, posteriorly barely contacting the medio-cranial extremities of the coracoid, cranially oriented. The coracoid foramen is very reduced, located in the center of the coracoid. The anterior (= primary) coracoid fenestra is not closed, the pericoracoid being too regressed to delimitate its anterior margin. The posterior (= secondary) coracoid fenestra is absent. Pentagonal with a process extending backward, the sternum is relatively well ossified, as long as wide, as wide as the interclavicle, without median sternal fontanel, and laterally connected to two pairs of sternal ribs. Absence of xiphisternum. **Forelimbs.** Forelimbs bones are almost completely regressed as it only subsists a pair of extremely reduced pear-shaped humerus articulating with the scapulocoracoid through a relatively well developed glenoid fossa. **Pelvic girdle.** Highly reduced, the pelvic girdle is composed of two separate elongated and curved hemipelves, without acetabulur depression. Pubis and ischium are laterally flattened and distally converging, forming the C-shaped bifurcated ventro-cranial end of each hemipelvis, whereas the ilium forms an elongated cigar-shaped dorso-caudal projection. **Hindlimbs.** Completely regressed, no vestigial bones.

***Voeltzkowia mobydick*** (UADBA R70487) **─ External features.** An elongated and slender, small-sized blind skink with two rounded flipper-like forelimbs, very short, slightly flattened, without any visible digits or claws; no visible external hindlimbs. **Pectoral girdle.** Complete and relatively well developed, the pectoral girdle is dorso-ventrally flattened and roughly rhomboidal, approximately as long as wide. Clavicles are strongly curved (S-shaped), flattened dorso-ventrally, with an enlarged and rounded proximal extremity, a narrow and pointed distal extremity, and a well-developed process at their mid-length posteriorly directed. The interclavicle is cruciform, with a round-ended anterior process approximately as long as the lateral processes which have narrow and pointed distal ends, and a round-ended posterior process about one and a half times longer. Suprascapulae are roughly triangular, more ossified medially than laterally. Scapula, coracoid and precoracoid are not distinct from each other, forming a continuous scapulocoracoid bone. The pericoracoid is extremely regressed, fragmented into several poorly ossified residues: two stripes separating the sternum from the coracoid, and two pairs of small rodlike structures, posteriorly barely contacting with the cranial extremities of the precoracoid and the coracoid, respectively, and anteriorly converging toward the anterior part of the interclavicle. The coracoid foramen is oval, almost open into the anterior coracoid fenestra. The anterior (= primary) coracoid fenestra is not completely closed, the pericoracoid being too reduced to delimitate its anterior margin. The posterior (= secondary) coracoid fenestra, located in the anterior part of the coracoid, may also be interpreted as a very thin and poorly ossified fossa rather than a true fenestra, as its margins are not clearly delimited from the surrounding osseus tissue. The pentagonal sternum is poorly ossified, as long as wide, as wide as the interclavicle, more ossified posteriorly than anteriorly, pierced by a large, round and median sternal fontanel in its posterior part, and laterally connected to two pairs of sternal ribs. The xiphisternum is “Y-shaped”, with three elongated rod-like processes: a median process connecting the posteriormost extremity of the sternum and two posterolaterally directed processes connecting a single pair of xiphisternal ribs. **Forelimbs.** Forelimbs are small but relatively well developed, with the exception of the autopodial bones which are significantly reduced in size and number. The humerus is relatively elongated, articulating with the scapulocoracoid through a relatively well developed glenoid fossa, and with enlarged proximal and distal ends twisted in relation to one another at an angle of approximately 90°. Ulna and radius are relatively reduced in comparison to the humerus, as they approximately represent only half of its length. Three globular elements can be assigned to the carpals: the largest, spherical and proximal is most likely the ulnar, and the two smaller probably represent distal carpals (possibly IV and V). Two elongated elements might represent metacarpals (possibly III and IV). There are no phalangeal bones. **Pelvic girdle.** Highly reduced, the pelvic girdle is composed of two separate, elongated and curved and rodlike hemipelves, without acetabulur depression. Pubis and ischium are apparently fused to form the anteroventral projection of each hemipelvis, distally compressed and curved, forming a trifurcated ventro-cranial end, whereas the ilium forms an elongated cigar-shaped dorso-caudal projection. **Hindlimbs.** Hindlimbs are absent, with the notable exception of two hardly distinguishable bony corpuscles probably representing rudiments of ancestral hindlimb bones, posterior to – and not in contact with – the pelvic girdle, floating freely below the cloacal vent. Less likely, these corpuscles may be interpreted as hemibacula (or hemibaubella), calcified structures present in the hemipenis (or hemiclitoris) of several distinct groups of squamate, except for the facts that (1) such structures are not present in the others species examined and (2) they would have been expected to lay deeper in the tail root, closer to the retractor muscle of the inverted hemipenis.
